# Short-Term Heart Rate Variability—Influence of Gender and Age in Healthy Subjects

**DOI:** 10.1371/journal.pone.0118308

**Published:** 2015-03-30

**Authors:** Andreas Voss, Rico Schroeder, Andreas Heitmann, Annette Peters, Siegfried Perz

**Affiliations:** 1 Department of Medical Engineering and Biotechnology, University of Applied Sciences Jena, Jena, Germany; 2 Institute of Epidemiology II, Helmholtz Zentrum München—German Research Center for Environmental Health, Neuherberg, Germany; 3 Institute for Biological and Medical Imaging, Helmholtz Zentrum München—German Research Center for Environmental Health, Neuherberg, Germany; Universidad Peruana de Ciencias Aplicadas (UPC), PERU

## Abstract

In the recent years, short-term heart rate variability (HRV) describing complex variations of beat-to-beat interval series that are mainly controlled by the autonomic nervous system (ANS) has been increasingly analyzed to assess the ANS activity in different diseases and under various conditions. In contrast to long-term HRV analysis, short-term investigations (<30 min) provide a test result almost immediately. Thus, short-term HRV analysis is suitable for ambulatory care, patient monitoring and all those applications where the result is urgently needed. In a previous study, we could show significant variations of 5-min HRV indices according to age in almost all domains (linear and nonlinear) in 1906 healthy subjects from the KORA S4 cohort. Based on the same group of subjects, general gender-related influences on HRV indices are to be determined in this study. Short-term 5-min HRV indices from linear time and frequency domain and from nonlinear methods (compression entropy, detrended fluctuation analysis, traditional and segmented Poincaré plot analysis, irreversibility analysis, symbolic dynamics, correlation and mutual information analysis) were determined from 782 females and 1124 males. First, we examined the gender differences in two age clusters (25–49 years and 50–74 years). Secondly, we investigated the gender-specific development of HRV indices in five age decade categories, namely for ages 25–34, 35–44, 45–54, 55–64 and 65–74 years. In this study, significant modifications of the indices according to gender could be obtained, especially in the frequency domain and correlation analyses. Furthermore, there were significant modifications according to age in nearly all of the domains. The gender differences disappeared within the last two age decades and the age dependencies disappeared in the last decade. To summarize gender and age influences need to be considered when performing HRV studies even if these influences only partly differ.

## Introduction

During the last few decades, particularly following the publication of the guidelines on heart rate variability (HRV) [1], the importance of HRV as a tool for assessing the autonomic nervous system activity in many different diseases and conditions has steadily increased. In addition to frequently used long-term HRV analysis, short-term HRV analysis has been increasingly applied in recent years due to its suitability for ambulatory care and short-term patient monitoring and due to the almost instant receiving of test results.

The HRV describes the complex variation of beat-to-beat intervals mainly controlled by the autonomic nervous system (ANS) through the interplay of sympathetic and parasympathetic neural activity at the sinus node [2]. In healthy subjects, typically showing a pronounced interindividual HRV [3], the dynamic cardiovascular control system is characterized by its ability to adapt to physiologic perturbations and changing conditions maintaining the cardiovascular homeostasis [4]. With aging or due to pathophysiological processes, changing structural factors (e.g. loss of sinoatrial pacemaker cells or of arterial distensibility) and/or functional changes (e.g. altered coupling between regulatory components) result in a reduction in overall HRV, a loss of complexity and altered fractal scaling. [4–7]. In general, the HRV is influenced by many several factors like chemical, hormonal and neural modulations, circadian changes, exercise, emotions, posture and preload. The adaptation of the heart rate to changing factors is carried out by the activity of different regulatory subsystems, i.e. activity of vasomotor and respiratory centers, of baroreflex and chemoreflex closed loop regulation, of cardiovascular reflexes mediated by vagal and sympathetic afferences, and of vascular- and thermo-regulation [7–9]. The variety of regulatory subsystems result in a complex linear and nonlinear cardiovascular control where the use of only classical linear signal analysis methods for HRV analysis is often inadequate [10–12]. Therefore, instead of applying only linear or only nonlinear methods, HRV analysis in both domains should be used in a complementary manner [7].

A limitation of short-term HRV analysis is the sparse availability of studies providing statistically relevant reference values for linear and, in particular, for nonlinear HRV indices from healthy subjects. To achieve a high degree of acceptance for HRV assessment in clinical practice, it is essential to determine age- and gender-related reference values for HRV indices from a healthy population on the basis of representative studies.

Several studies demonstrated age-related but also gender-related variation in long-term HRV significantly influencing the majority of investigated linear and nonlinear HRV indices [11, 13–15]. According to some of these studies, age- and gender-related changes in linear HRV indices of the short-time analysis were already found [16–25]. In general, a predominance of sympathetic tone over parasympathetic tone in males and vice versa in females was obtained. Furthermore, it was reported that autonomic activities diminish with age in both genders and that gender-related variation in parasympathetic regulation decreases after the age of 50 years [3, 16, 19, 20, 26–32].

Methods of nonlinear dynamics providing a considerably complex and extended analysis of long-term HRV gained considerable in importance in recent years [12, 33] and were partially used in the field of short-term HRV analysis [34–37]. However, valid information for short-term HRV indices of nonlinear dynamics has rarely been available due to the fact that it was derived from studies investigating only relatively small numbers of subjects [33, 38]. For these reasons it is of great importance and interest to ascertain the gender dependency of nonlinear dynamics HRV indices in general as well as the influence of gender on age-related changes in cardiac autonomic activity.

In a previous study, Voss et al. [39] investigated age dependencies of HRV indices from 5-min duration ECG recordings of 1906 healthy subjects of the population-based KORA (Cooperative Health Research in the Region Augsburg) S4 study [40]. The study’s results revealed significant variations for the majority of examined short-term (5-min) HRV indices according to age in both linear time and frequency domains and in the nonlinear dynamics domain. However, gender dependencies of HRV indices were not investigated.

This study has two major objectives: first objective is to ascertain the gender dependency of linear and particularly of nonlinear short-term HRV indices and second objective is to determine both age- and gender-related reference values for these short-term HRV indices based on a representative healthy population (1906 controls from KORA S4).

Compared to other studies in this field, strength of this study is the analysis of age- and gender-related HRV differences by the calculation of most published and international applied HRV indices of all domains based on a large population of healthy subjects. Until now, either studies that investigate several linear and/or nonlinear HRV indices on basis of small healthy subject groups or studies dealing with large subject groups but analyzed only linear or linear and only a few nonlinear HRV indices were performed. Hence, this study could serve as reference providing useful information about linear and particularly nonlinear indices for future studies in this field. Additionally, for the young and rather unexperienced scientists, the study results can emphasize the importance of an age- and gender-match of subject groups before performing a new study.

## Materials and Methods

### 1 Study Population

In this study, HRV analysis was performed on 5-min ECG recordings (lead II and lead V2 simultaneously, 500 Hz sample rate) obtained in supine position after a 5–10 minutes resting phase from the population-based KORA S4 database [40].

In a pre-selection process, from a total available number of 4107 subjects, 2201 subjects were excluded from the study. Based on carefully selected exclusion criteria and on the evaluation of an international used detailed standardized questionnaire (including medical history, lifestyle, and drug history etc.), with a high probability only healthy subjects were enrolled in this study. The self-rated health was accompanied by a standard 12 lead ECG. The remaining cohort of healthy subjects consisted of 782 women and 1124 men between the ages of 25 and 74 years (Table 1). Details about various exclusion criteria as presence of cardiac arrhythmia or artefacts, documented diseases, intake of heart rate affecting drugs, or pregnancy and the appropriate number of excluded subjects are provided in Table 2.

**Table 1:** Number of healthy female and male subjects in each age group (two and five age groups).

**Two age groups**	**Number of ♀ / ♂**	**Five age groups**	**Number of ♀ / ♂**
25–49 years	571 / 744	(1) 25–34 years	208 / 330
50–74 years	211 / 380	(2) 35–44 years	259 / 292
		(3) 45–54 years	158 / 235
		(4) 55–64 years	95 / 183
		(5) 65–74 years	62 / 84

**Table 2:** Exclusion criteria and the related number of excluded subjects (, with possible multiple assignments). In total 2201 subjects were excluded from the 4107 subjects leading to 1906 remaining healthy subjects.

**Exclusion criteria**	**Details**	**Number of excluded subjects**
presence of cardiac arrhythmia or artefacts	no sinus rhythm	242
	high percentage (> 10%) of ectopic beats or artefacts or poor signal quality	76
documented diseases	hypertension	965
	thyroid disease	486
	cancer	189
	peripheral vascular disease	163
	diabetes	155
	heart failure	137
	myocardial infarction	116
	angina pectoris	89
	apoplectic stroke	71
intake of heart rate affecting drugs	drug treated subjects	1349
	hormone replacement drug	338
	birth control pill	221
pregnancy	pregnant women	17

Approval by the ethical committee of the Bavarian Medical Association and the Bavarian commissioner for data protection and privacy was obtained before performing this study. In accordance with the recommendations of the Declaration of Helsinki, all subjects were informed about the study and gave their written informed consent prior to participation in the study.

### 2 Pre-processing

From the 5-min ECG recordings, time series of heart rate (tachogram) consisting of beat-to-beat intervals (RR-intervals) were extracted and visual inspected for correctness and abnormalities (arrhythmia, ectopic beats, artefacts or disturbances). In a next step, normal-to-normal (NN) interval time series were obtained by applying an adaptive filter [41] on the tachograms which detected and replaced the seldom occurring ectopic beats (ventricular or supra ventricular ectopic beats) and artefacts or disturbances with interpolated “normal” beats. Upon request, the author can supply the corresponding MATLAB-routine.

### 3 HRV Short-Term Analysis

In this study, a total of 68 HRV indices were determined by applying linear and nonlinear HRV analysis methods on the filtered tachograms. The applied methods of HRV analysis are briefly described as follows and explained in greater detail in [39].

#### 3.1 Time Domain (TD)

From time domain (TD) the following standard HRV indices were calculated according to the Task Force of the European Society of Cardiology and the North American Society of Pacing and Electrophysiology [1]:

- meanNN [ms]: mean value of NN interval time series
- sdNN [ms]: standard deviation of NN interval time series
- rmssd [ms]: square root of the mean squared differences of successive NN intervals
- sdaNN1 [ms]: standard deviation of the 1-min average of NN intervals
- pNN50 [%]: percentage derived by dividing the number of interval differences of successive NN intervals > 50 ms by the total number of NN intervals

Additionally, following HRV indices from time domain were estimated [42]:

- cvNN: ratio of sdNN divided by meanNN
- pNNI20 [%]: percentage of NN intervals differences < 20 ms
- shannon_h [bit]: Shannon Entropy (1) calculated on the basis of the class probabilities p_i_ (i = 1,…,n with n—number of classes) of the NN interval density distribution (class width of 8 ms resulting in a smoothed histogram suitable for HRV analysis [1])

$$\mathrm{shannon}\_h=-\sum_{i=1}^{n}p_{i}\;{\text{log}}_{2}\;p_{i}$$ (1)

renyi4 [bit]: Renyi entropy (2) according to Kurths et al. [43] was calculated from weighted probability distributions within a tachogram using a weighting coefficient of α = 4 that weights larger probabilities more than lower coefficients.

$$\mathrm{renyi}4=-\frac{1}{3}{\text{log}}_{2}\sum_{i=1}^{n}p_{i}^{4}$$ (2)

#### 3.2 Frequency Domain (FD)

From power spectra (Fast Fourier transformation using Blackman Harris window) of equidistant linear interpolated (10 Hz) tachograms (resampled to 2 Hz) the following frequency domain (FD) standard HRV indices [1] were determined:

- LF [ms²]: power in the „low“ frequency band 0.04–0.15 Hz
- HF [ms²]: power in the „high“ frequency band 0.15–0.4 Hz
- LF/HF: ratio between LF and HF
- P [ms^2^]: total power of the density spectra
- LFn: normalized LF power LFn = LF/(LF+HF)
- HFn: normalized HF power HFn = HF/(LF+HF).
- LF/P: ratio between LF and P
- HF/P: ratio between HF and P.

#### 3.3 Methods of Nonlinear Dynamics

According to Baumert et al. [44], the complexity of NN interval time series was quantified by using compression entropy (CE). CE is based on the Zip algorithm published by Ziv J and Lempel [45] and allows a lossless data compression using a sliding window technique, searching for and encoding matching sequences between the window and a look-ahead buffer. Depending on the window length w (= 3, 5, 7, 10, and 15) and the look-ahead buffer length b (= 3, 5, 7, 10, and 15) [46], the following index was estimated:

- H_c_^w,b^: ratio between the length M of the compressed time series and the length L of the original time series (3).

$$H_{c}^{w,b}=\frac{M}{L}$$ (3)

Detrended fluctuation analysis (DFA) introduced by Peng et al. [47] quantifies the fractal scaling properties of time series. To perform such an analysis, the NN interval time series is integrated y(k) (k = 1,…,N with N—length of time series) and divided into equal and non-overlapping segments of length n. In each segment, the local trend y_n_(k) is determined by least-squares fitting and subtracted from y(k). Finally, root-mean-square fluctuation values F(n) are calculated (4) and scaling exponents are estimated as the slope of the double-log plot of F(n) against n. Following scaling exponents were determined using segment length of n as recommended by Peng et al. [47]:

- α1: short-term fractal scaling exponent calculated over n = 4–16 beats
- α2: long-term fractal scaling exponent calculated over n = 16–64 beats.

$$F\left(n\right)=\sqrt{\frac{1}{N}\sum_{k=1}^{N}{\left[y(k)-y_{n}(k)\right]}^{2}}$$ (4)

The Poincaré plot analysis (PPA) is a geometrical and nonlinear quantitative method for the evaluation of the HRV dynamics [48]. Typically, from the cloud of points of a two-dimensional scatter plot where NN_n_ is plotted against NN_n+1_ (n = 1,…,N-1 and N—length of NN interval time series) the following linear PPA indices are calculated as proposed by Brennan et al. [49]:

- SD1 [ms]: standard deviation of the short-term NN interval variability (5)
- SD2 [ms]: standard deviation of the long-term NN interval variability (6)
- SD1/SD2: ratio between SD1 and SD2

$$SD1=\sqrt{\mathrm{variance}\left(\frac{NN_{n}-NN_{n+1}}{\sqrt{2}}\right)}$$ (5)

$$SD2=\sqrt{\mathrm{variance}\left(\frac{NN_{n}+NN_{n+1}}{\sqrt{2}}\right)}$$ (6)

Note, SD1 and rmssd (time domain) represent exactly the same information (correlation between both indices is one) even their values are different. SD1 values can be converted into rmssd values by multiplication of SD1 with the square root of two.

During segmented Poincaré plot analysis (SPPA), the cloud of points (see PPA) is rotated 45 degrees clockwise around the main focus of the cloud and segmented into 12x12 equal rectangles whose size depends on SD1 (height) and SD2 (width) as introduced by Voss et al. [50]. Based on the occurrence of points in each rectangle, a 12x12 probability matrix p_ij_ (row number: i = 1–12, column number: j = 1–12) was estimated from which the following SPPA indices were calculated:

- SPPA_r_i: single probability of each row (7)
- SPPA_c_j: single probability of each column (8)
- SPPA_entropy [bit]: Shannon entropy of the 12x12-probability matrix estimated according to Equation (1) using p_ij_ instead of p_i_.

$$SPPA\_r\_i=\sum_{j=1}^{12}p_{ij}$$ (7)

$$SPPA\_c\_j=\sum_{i=1}^{12}p_{ij}$$ (8)

Irreversibility analysis (IA) represents a relatively new approach that enables the detection of nonlinear irreversible dynamics characterized by temporal asymmetric patterns in a Poincaré plot [51]. In a Poincaré plot where NN_n_ is plotted against NN_n+τ_ (n = 1,…,N-τ; N—length of time series and τ—time lag (in this study τ = 1)), irreversibility is detectable if the points above the line of identity (ΔNN_n_^+^ = NN_n+τ_—NN_n_ > 0) and the points below the line of identity (ΔNN_n_^−^ = NN_n+τ_—NN_n_ < 0) are asymmetrically distributed. Three asymmetry (AS) indices were determined:

- AS1 [%]: Index introduced by Porta et al. [52] calculates the percentage of the number of points below the identity line M(ΔNN^−^) with respect to the number of points outside the identity line M(ΔNN ≠ 0) (9); values of AS1 that are not equal to 50% suggest irreversibility.

$$AS1=\frac{M\left(\Delta NN^{-}\right)}{M\left(\Delta NN\neq 0\right)}*100$$ (9)

AS2 [%]: Index introduced by Guzik et al. [53] quantifies the percentage of the sum of the squared distances (ΔNN_n_^+^ID) between the points above the identity line (ΔNN_n_^+^) and the identity line (ID) with respect to the sum of the squared distances (ΔNN_n_ID) between all points (ΔNN_n_) and the identity line (10); values of AS2 not equal to 50% suggest irreversibility.

$$AS2=\frac{\sum_{n=1}^{M\left(\Delta NN^{+}\right)}{\left(\Delta NN_{n}{}^{+}ID\right)}^{2}}{\sum_{n=1}^{M\left(\Delta NN\right)}{\left(\Delta NN_{n}ID\right)}^{2}}*100$$ (10)

- AS3: Index introduced by Ehlers et al. [54] assesses the distribution’s skewness of ∆NN with regard to the identity line (11); values of AS3 not equal to zero suggest irreversibility.

$$AS3=\frac{\sum_{n=1}^{M\left(\Delta NN\right)}{\left(\Delta NN_{n}\right)}^{3}}{{\left(\sum_{n=1}^{M\left(\Delta NN\right)}{\left(\Delta NN_{n}\right)}^{2}\right)}^{\frac{3}{2}}}$$ (11)

The application of the traditional symbolic dynamics (SD) method enables a simplified description of the dynamic of a system by coarse-graining of a time series [42, 43]. Following Voss et al. [42], NN interval time series were converted into strings of symbols of the alphabet A = {0, 1, 2, 3} according to transformation rules (12) with μ—mean of NN intervals and a—a special scaling parameter set to 0.1 determined as optimal value in [43].

$$\begin{array}{l} 0:\;\mu \;<\;NN_{n}\;<=\;\left(1+a\right)*\mu \\ 1:\;\left(1+a\right)*\mu \;<\;NN_{n}\;<\;\infty \\ 2:\;\left(1-a\right)*\mu \;<\;NN_{n}\;<=\;\mu \\ 3:\;0\;<\;NN_{n}\;<=\;\left(1-a\right)*\mu \end{array}$$ (12)

Subsequently, the symbol strings were transformed into word sequences, each consisting of three consecutive symbols. Following SD indices were determined using the probability distribution of the 64 (4^3^) possible word types:

- shannon_SD [bit]: Shannon entropy of the word distribution, a measure of complexity
- forbword: forbidden words (number of seldom (p < 0.001) or never occurring word types)
- wpsum02: relative portion of words consisting only of the symbols ‘0’ and ‘2’, a measure for decreased HRV
- wpsum13: relative portion of words consisting only of the symbols ‘1’ and ‘3’, a measure for increased HRV
- wsdvar: standard deviation of the word sequence
- phvar5: portion of high-variability patterns in the NN interval time series (> 5 ms)
- plvar20: portion of low-variability patterns in the NN interval time series (< 20 ms)
- fwrenyi025 [bit]: Renyi entropy of the word distribution with weight coefficient α = 0.25 weighting smaller probabilities
- fwrenyi4 [bit]: Renyi entropy of the word distribution with weight coefficient α = 4 weighting larger probabilities, Equation (2).

The short-term SD (STSD) by Porta et al. [9] was modified for the analysis of short-term tachograms (approx. 300 NN intervals). NN interval time series were converted into symbol strings with the alphabet B = {0, 1, 2, 3, 4, 5} based on six equally distributed class ranges. Then, word sequences each consisting of three successive symbols were generated. Both, for the number of possible symbols (quantification level) and the number of the word sequence length small values were chosen to keep a low number of possible pattern ensuring the reliability of the probability density function for series with approx. 300 NN intervals. By evaluating the probability distribution of the 216 (6^3^) possible word pattern types, the following STSD indices were calculated by grouping individual word patterns into various pattern families [9]:

- ST_MP: portion of missing patterns
- ST_0V: portion of words of the pattern family 0V (three successive symbols are equal, e.g. ‘111’ or ‘444’)
- ST_1V: portion of words of the pattern family 1V (two successive symbols are equal and the remaining one symbol differs, e.g. ‘344’ or ‘331’).
- ST_2V: portion of words of the pattern family 2V (two variations in the word, e.g. ‘1 2 3’ or ‘5 3 4’)
- ST_2LV: portion of words of the pattern family 2LV (three successive symbols form an ascending or a descending ramp, e.g. ‘1 2 4’ or ‘5 4 3’)
- ST_2UV: portion of words of the pattern family 2UV (second symbol is larger or smaller than the other two symbols forming a peak or valley, e.g. ‘3 4 3’ or ‘3 0 2’).

In addition, STSD indices describing further pattern families were determined as introduced by Heitmann et al. [55]:

- ST_ASC: portion of words of the pattern family ASC (where three successive symbols form an ascending ramp, e.g. ‘234’ or ‘012’)
- ST_DESC: portion of words of the pattern family DESC (where three successive symbols form a descending ramp, e.g. ‘432’ or ‘210’)
- ST_PEAK: portion of words of the pattern family PEAK (the second symbol is larger than the other two symbols forming a peak, e.g. ‘121’ or ‘243’)
- ST_VAL: portion of words of the pattern family VAL (the second symbol is smaller than the other two symbols forming a valley, e.g. ‘212’ or ‘312’).

Auto-correlation (ACOR) and auto-mutual information (AMI) analysis is used in general to discover statistical dependencies within one time series [56, 57]. In relation to HRV analysis, the ACOR function is obtained when the NN interval time series is shifted against itself determining for each shift τ (which was set to 1) a respective correlation coefficient. To get the AMI function, introduced by Shannon [58], for each τ one mutual information coefficient instead of the correlation coefficient is calculated. The mutual information coefficient based on the determination of the marginal Shannon entropies as well as the joint Shannon entropy. For the estimation of the Shannon entropies, nine classes were used to create the NN interval density distribution. The number of classes was calculated using the Sturges’ criterion (number of classes = 1 + 3.32*log(N), N…number of NN intervals) [59].

From the resulting ACOR function, the following linear indices describing the interaction strength within a time series (x2peakrrcor and y2peakrrcor) and the temporal predictability of a time series (a21rrcor, a31rrcor, amax21rrcor) were extracted:

- x2peakrrcor [beats]: location of the highest auxiliary maximum of the ACOR function
- y2peakrrcor: amplitude of the highest auxiliary maximum of the ACOR function
- a21rrcor: slope of the ACOR function from the maximum ACOR coefficient (τ = 0) to the subsequent ACOR value (τ = 1)
- a31rrcor: slope of the ACOR function from the maximum ACOR coefficient (τ = 0) to the second succeeding ACOR value (τ = 2)
- amax21rrcor: slope of the ACOR function from the maximum ACOR coefficient (τ = 0) to the highest auxiliary ACOR maximum.

From the resulting AMI function, indices characterizing the linear and/or nonlinear interaction strength and the temporal predictability of a time series were calculated:

- x2peakrr [beats]: location of the highest auxiliary maximum of the AMI function
- y2peakrr: amplitude of the highest auxiliary maximum of the AMI function
- a21rr [bit/beat]: slope of the AMI function from the maximum AMI coefficient (τ = 0) to the subsequent AMI value (τ = 1)
- a31rr [bit/beat]: slope of the AMI function from the maximum AMI coefficient (τ = 0) to the second succeeding AMI value (τ = 2).
- amax21rr: slope of the AMI function from the maximum AMI coefficient (τ = 0) to the highest auxiliary AMI maximum.

### 4 Statistical analyses

The complete univariate statistical analysis was performed with IBM SPSS Statistics 19.0 for Windows. Descriptive statistics were performed to calculate mean values, standard deviations, median values and interquartile ranges for all HRV indices in both age clusters (using two and five age categories respectively), subdivided into men and women (Table 1). In order to facilitate comparisons to other findings within the field where the published papers deals almost exclusively with mean values, it was decided to present mean values and standard deviations in this manuscript. Median values and interquartile ranges can be found as supplementary data in the supporting information section (Tables A-F in S1 Complementary Statistics). To test the hypotheses that (1) age and gender influence short-term analyses over all domains (linear and nonlinear), and that (2) age and gender dependencies of individual HRV indices diminish with ageing, the following statistical tests were designed.

#### 4.1 Test I—General age and gender dependency

In Test I, the subjects of two age groups 25–49 years and 50–74 years were each subdivided into two female and male groups (YF—young females, EF—elderly females, YM—young males, and EM—elderly males). Age and gender dependencies of HRV indices were assessed using the Mann-Whitney U test (most of the 65 investigated HRV indices were not normally distributed as proved by the Kolmogorov-Smirnov test) for two independent samples. To investigate the dependence on age in both gender groups, the HRV indices of the age groups 25–49 years were statistically compared to the HRV indices of the age groups 50–74 years (tests: YF vs. EF and YM vs. EM). Gender dependency was analyzed by comparing the HRV indices of both gender groups separately for the age groups (tests: YF vs. YM and EF vs. EM).

#### 4.2 Test II—Gender-specific development of age dependency

In Test II, HRV indices of five age groups 25–34, 35–44, 45–54, 55–64, and 65–74 years were compared to each other in terms of female and male subjects respectively. For these comparisons, we applied the Kruskal-Wallis test with subsequent Mann-Whitney U tests. Additionally, the gender dependency in each age decade was investigated (Mann-Whitney U test) to determine if gender-induced differences of HRV indices changed with ageing.

We used the following significance levels:

− p≥0.01: not significant—NS
− p<0.01: significant on level * (lowest)
− p<0.0007: significant on level ** (Bonferroni criterion)
− p<10–10: significant on level *** (high)
− p<10–20: significant on level **** (highest)

## Results

Descriptive statistics and the results of Test I and Test II are presented in Tables 3–4 and Tables 5–10 respectively. Tables 5–8 contain both the descriptive statistics and the results of individual comparisons between the five investigated age decades separated according to gender. Univariate test results considering the gender dependency in five different age decades are shown in Tables 9–10.

**Table 3:** Descriptive statistics and results of Mann-Whitney U tests with respect to linear HRV indices according to Tests I comparing each two age clustered (25–49 years and 50–74 years) female and/or male subject groups.

		**Mean ± standard deviation**				**Mann-Whitney U test**			
**MA**	**HRV-index**	**YF**	**YM**	**EF**	**EM**	**YM-EM**	**YF-EF**	**YF-YM**	**EF-EM**
	**Number (N =)**	**571**	**744**	**211**	**380**				
**TD**	meanNN	901 ± 117	930 ± 133	880 ± 115	911 ± 128	NS	NS	**	*
	sdNN	44.9 ± 19.2	45.8 ± 18.8	31.6 ± 13.6	33.0 ± 14.8	****	****	NS	NS
	cvNN	0.05 ± 0.02	0.05 ± 0.02	0.04 ± 0.01	0.04 ± 0.01	****	****	NS	NS
	sdaNN1	18.1 ± 13.2	18.2 ± 11.4	14.3 ± 8.3	15.1 ± 9.7	**	**	NS	NS
	rmssd	36.5 ± 20.1	34.0 ± 18.3	22.0 ± 13.2	20.5 ± 11.0	****	****	NS	NS
	pNN50	0.17 ± 0.18	0.15 ± 0.16	0.05 ± 0.09	0.04 ± 0.07	****	****	NS	NS
	pNNl20	0.49 ± 0.21	0.52 ± 0.22	0.71 ± 0.21	0.74 ± 0.19	****	****	NS	NS
	renyi4	3.87 ± 0.54	3.88 ± 0.55	3.34 ± 0.55	3.39 ± 0.58	****	****	NS	NS
	shannon_h	4.30 ± 0.53	4.32 ± 0.53	3.79 ± 0.55	3.84 ± 0.57	****	****	NS	NS
**FD**	LF	159 ± 181	203 ± 262	66 ± 83	84 ± 115	****	****	**	*
	HF	125 ± 147	101 ± 143	41.4 ± 72.1	29.5 ± 36.6	****	****	*	*
	P	531 ± 638	533 ± 534	247 ± 282	284 ± 313	****	****	NS	NS
	LF/HF	2.09 ± 2.05	3.33 ± 3.47	2.75 ± 2.93	4.29 ± 4.06	**	*	****	**
	LF/P	0.31 ± 0.14	0.38 ± 0.16	0.28 ± 0.13	0.31 ± 0.15	**	NS	****	*
	HF/P	0.24 ± 0.15	0.19 ± 0.13	0.17 ± 0.12	0.12 ± 0.10	****	**	**	**
	LFn	0.58 ± 0.19	0.67 ± 0.17	0.63 ± 0.18	0.72 ± 0.17	**	*	****	**
	HFn	0.42 ± 0.19	0.33 ± 0.17	0.37 ± 0.18	0.28 ± 0.17	**	*	****	**

Abbreviations: YF, young females; YM, young males; EF, elderly females; EM, elderly males; MA, method of analysis; TD, time domain; FD, frequency domain; level of significance:

****p<10^−20^,

***p<10^−10^,

**p<Bonferroni criterion = 0.0007,

*p<0.01, NS, no significance.

**Table 4:** Descriptive statistics and results of Mann-Whitney U tests with respect to nonlinear HRV indices according to Tests I comparing each two age clustered (25–49 years and 50–74 years) female and/or male subject groups.

		**Mean ± standard deviation**				**Mann-Whitney U test**			
**MA**	**HRV-index**	**YF**	**YM**	**EF**	**EM**	**YM-EM**	**YF-EF**	**YF-YM**	**EF-EM**
	**Number (N =)**	**571**	**744**	**211**	**380**				
**SD**	shannon_SD	3.08 ± 0.49	2.99 ± 0.47	2.50 ± 0.52	2.44 ± 0.49	****	****	*	NS
	forbword	26.9 ± 11.0	28.9 ± 9.9	37.0 ± 10.2	38.5 ± 8.8	****	****	*	NS
	wpsum02	0.49 ± 0.25	0.52 ± 0.23	0.73 ± 0.20	0.73 ± 0.20	****	****	NS	NS
	wpsum13	0.09 ± 0.09	0.10 ± 0.09	0.05 ± 0.06	0.07 ± 0.08	****	****	NS	NS
	wsdvar	1.24 ± 0.45	1.24 ± 0.44	0.85 ± 0.43	0.88 ± 0.48	****	****	NS	NS
	phvar5	0.44 ± 0.20	0.42 ± 0.20	0.25 ± 0.18	0.23 ± 0.17	****	****	NS	NS
	plvar20	0.09 ± 0.18	0.12 ± 0.22	0.31 ± 0.32	0.35 ± 0.32	****	****	*	NS
	fwrenyi025	3.43 ± 0.37	3.37 ± 0.35	3.02 ± 0.42	2.97 ± 0.40	****	****	*	NS
	fwrenyi4	2.49 ± 0.61	2.36 ± 0.58	1.82 ± 0.54	1.75 ± 0.50	****	****	**	NS
**DFA**	α1	0.92 ± 0.23	0.98 ± 0.22	1.06 ± 0.24	1.13 ± 0.23	****	****	**	*
	α2	0.91 ± 0.20	0.87 ± 0.22	0.98 ± 0.17	0.97 ± 0.20	****	**	*	NS
**CE**	H_c_^3,3^	0.78 ± 0.08	0.77 ± 0.09	0.68 ± 0.09	0.67 ± 0.09	****	****	NS	NS
**STSD**	ST_MP	0.70 ± 0.08	0.72 ± 0.07	0.74 ± 0.07	0.75 ± 0.07	**	**	**	NS
	ST_0V	0.06 ± 0.07	0.05 ± 0.07	0.04 ± 0.07	0.03 ± 0.06	****	****	**	NS
	ST_1V	0.46 ± 0.12	0.51 ± 0.13	0.54 ± 0.14	0.55 ± 0.15	**	****	****	NS
	ST_2V	0.27 ± 0.10	0.24 ± 0.10	0.20 ± 0.10	0.20 ± 0.11	****	****	**	NS
	ST_INC	0.09 ± 0.05	0.09 ± 0.05	0.06 ± 0.04	0.07 ± 0.04	****	**	NS	NS
	ST_DESC	0.08 ± 0.04	0.08 ± 0.04	0.06 ± 0.04	0.06 ± 0.04	**	****	NS	NS
	ST_PEAK	0.15 ± 0.05	0.13 ± 0.05	0.14 ± 0.05	0.15 ± 0.06	*	NS	*	NS
	ST_VAL	0.16 ± 0.06	0.14 ± 0.05	0.16 ± 0.05	0.15 ± 0.05	*	NS	****	NS
	ST_2LV	0.16 ± 0.07	0.17 ± 0.08	0.12 ± 0.07	0.13 ± 0.08	****	****	NS	NS
	ST_2UV	0.31 ± 0.09	0.27 ± 0.08	0.30 ± 0.09	0.29 ± 0.09	**	NS	****	NS
**PPA**	SD1	25.8 ± 14.2	24.1 ± 13.0	15.5 ± 9.3	14.5 ± 7.8	****	****	NS	NS
	SD2	57.5 ± 24.4	59.7 ± 24.2	41.3 ± 17.6	43.9 ± 20.1	****	****	NS	NS
	SD1/SD2	0.45 ± 0.16	0.40 ± 0.13	0.38 ± 0.15	0.34 ± 0.14	****	****	**	*
**SPPA**	SPPA_c_4	2.14 ± 1.23	2.06 ± 1.22	2.09 ± 1.17	2.29 ± 1.35	*	NS	NS	NS
	SPPA_c_5	12.5 ± 3.1	12.4 ± 3.3	12.0 ± 3.4	12.1 ± 3.4	NS	NS	NS	NS
	SPPA_c_6	34.5 ± 4.9	34.6 ± 5.1	34.3 ± 5.1	33.7 ± 5.3	*	NS	NS	NS
	SPPA_c_7	35.1 ± 5.4	35.3 ± 5.5	36.2 ± 6.2	36.4 ± 6.4	NS	NS	NS	NS
	SPPA_c_8	13.4 ± 2.4	13.2 ± 2.5	12.9 ± 2.7	13.2 ± 2.7	NS	NS	NS	NS
	SPPA_c_9	1.84 ± 1.11	1.88 ± 1.13	1.87 ± 1.30	1.66 ± 1.24	*	NS	NS	NS
	SPPA_r_4	1.73 ± 0.97	2.08 ± 0.98	1.83 ± 0.93	1.87 ± 0.86	*	NS	**	NS
	SPPA_r_5	13.5 ± 2.7	12.5 ± 2.8	12.5 ± 3.2	11.8 ± 3.4	*	*	****	NS
	SPPA_r_6	35.6 ± 4.1	34.6 ± 4.3	35.0 ± 4.3	36.0 ± 4.3	**	NS	**	*
	SPPA_r_7	31.9 ± 5.1	34.6 ± 5.2	35.1 ± 5.2	35.3 ± 4.8	NS	****	****	NS
	SPPA_r_8	15.2 ± 3.0	14.0 ± 3.2	13.2 ± 3.4	12.5 ± 3.4	**	****	****	NS
	SPPA_r_9	1.66 ± 0.81	1.53 ± 0.82	1.75 ± 1.03	1.79 ± 0.84	**	NS	*	NS
	SPPA_entropy	4.03 ± 0.10	4.01 ± 0.11	3.99 ± 0.15	3.98 ± 0.16	*	NS	NS	NS
**IA**	AS1	49.3 ± 3.5	50.7 ± 3.7	50.5 ± 3.5	50.0 ± 2.9	*	**	****	NS
	AS2	51.3 ± 5.9	53.5 ± 6.3	53.3 ± 7.4	53.0 ± 7.0	NS	*	****	NS
	AS3	0.01 ± 0.07	0.03 ± 0.06	0.04 ± 0.11	0.04 ± 0.1	**	**	**	NS
**ACOR and AMI**	a21rr	−2.38 ± 0.31	−2.26 ± 0.32	−2.15 ± 0.33	−2.07 ± 0.34	****	****	****	NS
	a31rr	−1.31 ± 0.12	−1.28 ± 0.13	−1.21 ± 0.14	−1.19 ± 0.15	****	****	**	NS
	x2peakrr	5.73 ± 1.99	6.31 ± 2.22	6.74 ± 2.42	7.40 ± 2.77	***	**	**	NS
	y2peakrr	0.46 ± 0.15	0.42 ± 0.13	0.49 ± 0.17	0.47 ± 0.18	**	**	**	NS
	amax21rr	−0.50 ± 0.15	−0.46 ± 0.14	−0.41 ± 0.13	−0.39 ± 0.14	****	****	**	NS
	a21rrcor	−0.34 ± 0.19	−0.29 ± 0.15	−0.26 ± 0.18	−0.22 ± 0.16	****	****	**	NS
	a31rrcor	−0.31 ± 0.13	−0.29 ± 0.12	−0.22 ± 0.11	−0.20 ± 0.09	****	****	*	*
	x2peakrrcor	5.96 ± 3.32	7.56 ± 3.80	8.28 ± 5.51	9.78 ± 5.78	**	**	****	**
	y2peakrrcor	0.42 ± 0.18	0.35 ± 0.17	0.39 ± 0.23	0.33 ± 0.22	NS	NS	****	*
	amax21rrcor	−0.12 ± 0.05	−0.11 ± 0.05	−0.09 ± 0.05	−0.09 ± 0.05	**	****	**	NS

Abbreviations: YF, young females; YM, young males; EF, elderly females; EM, elderly males; MA, method of analysis; SD, symbolic dynamic; DFA, detrended fluctuation analysis; CE, compression entropy; STSD, short-term symbolic dynamic; PPA, Poincaré plot analysis; SPPA, segmented Poincaré plot analysis; IA, irreversibility analysis; ACOR and AMI, auto-correlation and auto-mutual information; level of significance:

****p<10^−20^,

***p<10^−10^,

**p<Bonferroni criterion = 0.0007,

*p<0.01, NS, no significance.

**Table 5:** Descriptive statistics and results of Kruskal-Wallis test followed by Mann-Whitney U tests with respect to linear HRV indices comparing each two age decades in females (Tests II, comparison of five different age decades: 1 = 25–34, 2 = 35–44, 3 = 45–54, 4 = 55–64 and 5 = 65–74 years).

		**1 (N = 208)**	**2 (N = 259)**	**3 (N = 158)**	**4 (N = 95)**	**5 (N = 62)**	**1–5**	**1 vs. 2**	**2 vs. 3**	**3 vs. 4**	**4 vs. 5**
**MA**	**HRV-index**	**Mean ± standard deviation**					**KW test**	**Mann-Whitney U tests**			
**TD**	meanNN	900 ± 116	903 ± 122	903 ± 109	868 ± 118	873 ± 110	NS	NS	NS	*	NS
	sdNN	48.7 ± 19.0	45.4 ± 20.5	36.9 ± 13.8	30.6 ± 12.4	27.8 ± 11.8	****	NS	**	**	NS
	cvNN	0.05 ± 0.02	0.05 ± 0.02	0.04 ± 0.01	0.04 ± 0.01	0.03 ± 0.01	****	*	**	*	NS
	sdaNN1	17.5 ± 10.8	19.4 ± 16.0	16.4 ± 8.8	13.7 ± 7.9	12.9 ± 7.5	**	NS	NS	*	NS
	rmssd	42.9 ± 22.8	35.4 ± 18.5	26.3 ± 13.6	21.4 ± 11.9	19.1 ± 11.8	****	*	**	*	NS
	pNN50	0.23 ± 0.20	0.16 ± 0.17	0.08 ± 0.12	0.05 ± 0.08	0.04 ± 0.06	****	**	**	*	NS
	pNNl20	0.42 ± 0.20	0.49 ± 0.21	0.61 ± 0.20	0.71 ± 0.20	0.78 ± 0.19	****	**	**	*	NS
	renyi4	4.00 ± 0.52	3.87 ± 0.54	3.60 ± 0.50	3.29 ± 0.53	3.17 ± 0.53	****	*	**	**	NS
	shannon_h	4.43 ± 0.50	4.31 ± 0.53	4.04 ± 0.49	3.76 ± 0.54	3.62 ± 0.53	****	*	**	**	NS
**FD**	LF	184 ± 199	161 ± 177	107 ± 136	57 ± 59	45 ± 56	****	NS	*	**	NS
	HF	161 ± 167	121 ± 145	62 ± 83	35 ± 53	29 ± 38	****	**	**	**	NS
	P	580 ± 488	566 ± 811	366 ± 343	195 ± 166	188 ± 212	****	NS	*	**	NS
	LF/HF	1.75 ± 1.78	2.21 ± 2.16	2.43 ± 1.99	2.87 ± 3.32	2.97 ± 3.18	**	*	NS	NS	NS
	LF/P	0.32 ± 0.14	0.31 ± 0.14	0.30 ± 0.15	0.29 ± 0.13	0.26 ± 0.11	NS	NS	NS	NS	NS
	HF/P	0.28 ± 0.15	0.23 ± 0.14	0.18 ± 0.12	0.18 ± 0.12	0.17 ± 0.12	***	*	*	NS	NS
	LFn	0.54 ± 0.18	0.59 ± 0.19	0.63 ± 0.17	0.64 ± 0.18	0.63 ± 0.19	**	*	NS	NS	NS
	HFn	0.46 ± 0.18	0.41 ± 0.19	0.37 ± 0.17	0.36 ± 0.18	0.37 ± 0.19	**	*	NS	NS	NS

Abbreviations: MA, method of analysis; TD, time domain; FD, frequency domain; N: number of subjects; KW test: Kruskal-Wallis test; level of significance:

****p<10^−20^,

***p<10^−10^,

**p<Bonferroni criterion = 0.0007,

*p<0.01, NS, no significance.

**Table 6:** Descriptive statistics and results of Kruskal-Wallis test followed by Mann-Whitney U tests with respect to nonlinear HRV indices comparing each two age decades in females (Tests II, comparison of five different age decades: 1 = 25–34, 2 = 35–44, 3 = 45–54, 4 = 55–64 and 5 = 65–74 years).

		**1 (N = 208)**	**2 (N = 259)**	**3 (N = 158)**	**4 (N = 95)**	**5 (N = 62)**	**1–5**	**1 vs. 2**	**2 vs. 3**	**3 vs. 4**	**4 vs. 5**
**MA**	**HRV-index**	**Mean ± standard deviation**					**KW test**	**Mann-Whitney U tests**			
**SD**	shannon_SD	3.26 ± 0.44	3.05 ± 0.46	2.76 ± 0.48	2.51 ± 0.49	2.30 ± 0.53	****	**	**	**	NS
	forbword	22.7 ± 10.8	27.5 ± 10.3	33.8 ± 9.5	36.9 ± 10.1	39.6 ± 10.0	****	*	**	**	NS
	wpsum02	0.41 ± 0.24	0.49 ± 0.24	0.64 ± 0.21	0.74 ± 0.18	0.79 ± 0.19	****	*	**	*	NS
	wpsum13	0.10 ± 0.09	0.09 ± 0.10	0.07 ± 0.06	0.05 ± 0.06	0.04 ± 0.07	***	NS	*	*	NS
	wsdvar	1.35 ± 0.43	1.25 ± 0.44	1.02 ± 0.42	0.84 ± 0.40	0.71 ± 0.43	****	*	**	**	NS
	phvar5	0.51 ± 0.19	0.44 ± 0.19	0.33 ± 0.19	0.25 ± 0.18	0.19 ± 0.17	****	**	**	*	NS
	plvar20	0.05 ± 0.13	0.09 ± 0.18	0.16 ± 0.23	0.32 ± 0.31	0.42 ± 0.35	****	**	**	**	NS
	fwrenyi025	3.56 ± 0.33	3.42 ± 0.34	3.19 ± 0.37	3.03 ± 0.40	2.88 ± 0.45	****	**	**	**	NS
	fwrenyi4	2.71 ± 0.58	2.46 ± 0.58	2.11 ± 0.56	1.81 ± 0.52	1.62 ± 0.51	****	**	**	**	NS
**DFA**	α1	0.85 ± 0.21	0.93 ± 0.22	1.02 ± 0.24	1.04 ± 0.23	1.10 ± 0.26	***	**	*	NS	NS
	α2	0.88 ± 0.22	0.92 ± 0.20	0.94 ± 0.18	0.98 ± 0.17	0.97 ± 0.18	**	NS	NS	NS	NS
**CE**	H_c_^3,3^	0.80 ± 0.07	0.78 ± 0.08	0.73 ± 0.08	0.68 ± 0.09	0.65 ± 0.10	****	*	**	*	NS
**STSD**	ST_MP	0.68 ± 0.08	0.70 ± 0.07	0.73 ± 0.07	0.74 ± 0.07	0.76 ± 0.07	***	*	**	NS	NS
	ST_0V	0.07 ± 0.07	0.06 ± 0.07	0.05 ± 0.07	0.04 ± 0.07	0.03 ± 0.06	**	*	NS	NS	NS
	ST_1V	0.44 ± 0.11	0.47 ± 0.12	0.51 ± 0.14	0.53 ± 0.14	0.55 ± 0.16	**	*	*	NS	NS
	ST_2V	0.29 ± 0.10	0.27 ± 0.10	0.23 ± 0.10	0.2 ± 0.10	0.19 ± 0.11	***	NS	**	NS	NS
	ST_INC	0.09 ± 0.05	0.09 ± 0.04	0.08 ± 0.04	0.06 ± 0.04	0.06 ± 0.04	**	NS	*	NS	NS
	ST_DESC	0.08 ± 0.04	0.08 ± 0.04	0.07 ± 0.04	0.06 ± 0.04	0.06 ± 0.05	**	NS	NS	*	NS
	ST_PEAK	0.15 ± 0.05	0.15 ± 0.05	0.14 ± 0.05	0.15 ± 0.06	0.14 ± 0.05	NS	NS	NS	NS	NS
	ST_VAL	0.17 ± 0.05	0.16 ± 0.06	0.16 ± 0.05	0.16 ± 0.05	0.17 ± 0.06	NS	NS	NS	NS	NS
	ST_2LV	0.17 ± 0.07	0.17 ± 0.07	0.15 ± 0.07	0.12 ± 0.07	0.12 ± 0.08	***	NS	*	*	NS
	ST_2UV	0.32 ± 0.08	0.30 ± 0.09	0.29 ± 0.08	0.30 ± 0.09	0.31 ± 0.10	NS	NS	NS	NS	NS
**PPA**	SD1	30.3 ± 16.1	25.1 ± 13.1	18.6 ± 9.6	15.1 ± 8.4	13.2 ± 8.2	****	*	**	*	NS
	SD2	61.2 ± 23.2	58.5 ± 27.0	48.3 ± 17.6	40.0 ± 16.4	36.0 ± 14.7	****	NS	*	**	NS
	SD1/SD2	0.49 ± 0.16	0.43 ± 0.15	0.39 ± 0.13	0.38 ± 0.16	0.36 ± 0.15	***	*	*	NS	NS
**SPPA**	SPPA_c_4	2.12 ± 1.20	2.18 ± 1.24	2.08 ± 1.25	2.25 ± 1.18	1.91 ± 1.13	NS	NS	NS	NS	NS
	SPPA_c_5	12.5 ± 2.8	12.5 ± 3.2	12.1 ± 3.4	11.5 ± 3.2	13.0 ± 3.6	NS	NS	NS	NS	NS
	SPPA_c_6	35.1 ± 4.7	34.1 ± 5.0	34.2 ± 4.6	34.4 ± 5.3	34.1 ± 5.2	NS	*	NS	NS	NS
	SPPA_c_7	34.1 ± 5.3	35.5 ± 5.1	36.3 ± 6.0	36.5 ± 6.4	35.2 ± 5.8	**	**	NS	NS	NS
	SPPA_c_8	13.7 ± 2.2	13.4 ± 2.4	12.8 ± 2.6	12.7 ± 2.9	13.3 ± 2.4	NS	NS	NS	NS	NS
	SPPA_c_9	1.97 ± 1.09	1.76 ± 1.12	1.80 ± 1.12	1.81 ± 1.49	1.95 ± 1.09	NS	NS	NS	NS	NS
	SPPA_r_4	1.67 ± 0.92	1.74 ± 0.99	1.81 ± 1.03	1.92 ± 0.84	1.74 ± 0.92	NS	NS	NS	NS	NS
	SPPA_r_5	13.9 ± 2.5	13.5 ± 2.8	13.0 ± 3.0	12.5 ± 3.2	11.9 ± 3.1	**	NS	NS	NS	NS
	SPPA_r_6	35.5 ± 4.4	35.5 ± 3.8	35.4 ± 4.0	34.9 ± 4.2	35.5 ± 4.3	NS	NS	NS	NS	NS
	SPPA_r_7	31.2 ± 5.1	32.3 ± 5.2	33.2 ± 4.8	35.0 ± 4.9	36.0 ± 5.7	***	NS	NS	NS	NS
	SPPA_r_8	15.8 ± 2.7	14.9 ± 3.1	14.4 ± 2.8	13.3 ± 3.5	12.1 ± 3.5	***	*	NS	NS	NS
	SPPA_r_9	1.58 ± 0.81	1.71 ± 0.82	1.68 ± 0.78	1.71 ± 1.06	1.91 ± 1.18	NS	NS	NS	NS	NS
	SPPA_entropy	4.04 ± 0.07	4.02 ± 0.08	4.01 ± 0.16	3.99 ± 0.15	3.99 ± 0.14	NS	NS	NS	NS	NS
**IA**	AS1	49.2 ± 3.8	49.3 ± 3.5	49.6 ± 3.2	50.4 ± 3.1	50.8 ± 3.8	*	NS	NS	NS	NS
	AS2	51.2 ± 5.5	51.4 ± 5.8	51.8 ± 6.9	53.0 ± 6.6	54.4 ± 9.0	NS	NS	NS	NS	NS
	AS3	0.01 ± 0.05	0.02 ± 0.06	0.02 ± 0.10	0.04 ± 0.11	0.06 ± 0.13	NS	NS	NS	NS	NS
**ACOR and AMI**	a21rr	−2.47 ± 0.29	−2.36 ± 0.30	−2.23 ± 0.32	−2.16 ± 0.35	−2.08 ± 0.32	****	*	**	NS	NS
	a31rr	−1.35 ± 0.10	−1.30 ± 0.12	−1.26 ± 0.13	−1.21 ± 0.15	−1.18 ± 0.14	****	*	*	NS	NS
	x2peakrr	5.32 ± 1.53	5.84 ± 2.09	6.49 ± 2.38	6.53 ± 2.38	6.98 ± 2.48	**	NS	NS	NS	NS
	y2peakrr	0.45 ± 0.14	0.47 ± 0.16	0.46 ± 0.14	0.50 ± 0.18	0.50 ± 0.18	NS	NS	NS	NS	NS
	amax21rr	−0.54 ± 0.14	−0.49 ± 0.15	−0.44 ± 0.14	−0.43 ± 0.14	−0.39 ± 0.12	***	*	*	NS	NS
	a21rrcor	−0.40 ± 0.20	−0.33 ± 0.18	−0.27 ± 0.16	−0.27 ± 0.19	−0.25 ± 0.17	***	*	*	NS	NS
	a31rrcor	−0.36 ± 0.14	−0.30 ± 0.13	−0.26 ± 0.11	−0.22 ± 0.10	−0.20 ± 0.10	****	**	*	NS	NS
	x2peakrrcor	5.34 ± 2.71	6.22 ± 3.60	6.78 ± 3.71	8.42 ± 5.96	8.95 ± 5.89	**	NS	NS	NS	NS
	y2peakrrcor	0.42 ± 0.16	0.42 ± 0.19	0.41 ± 0.19	0.39 ± 0.24	0.39 ± 0.24	NS	NS	NS	NS	NS
	amax21rrcor	−0.13 ± 0.05	−0.11 ± 0.05	−0.10 ± 0.05	−0.10 ± 0.06	−0.09 ± 0.06	***	*	NS	NS	NS

Abbreviations: MA, method of analysis; SD, symbolic dynamic; DFA, detrended fluctuation analysis; CE, compression entropy; STSD, short-term symbolic dynamic; PPA, Poincaré plot analysis; SPPA, segmented Poincaré plot analysis; IA, irreversibility analysis; ACOR and AMI, auto-correlation and auto-mutual information; N: number of subjects; KW test: Kruskal-Wallis test; level of significance:

****p<10^−20^,

***p<10^−10^,

**p<Bonferroni criterion = 0.0007,

*p<0.01, NS, no significance.

**Table 7:** Descriptive statistics and results of Kruskal-Wallis test followed by Mann-Whitney U tests with respect to linear HRV indices comparing each two age decades in males (Tests II, comparison of five different age decades: 1 = 25–34, 2 = 35–44, 3 = 45–54, 4 = 55–64 and 5 = 65–74 years).

		**1 (N = 330)**	**2 (N = 292)**	**3 (N = 235)**	**4 (N = 183)**	**5 (N = 84)**	**1–5**	**1 vs. 2**	**2 vs. 3**	**3 vs. 4**	**4 vs. 5**
**MA**	**HRV-index**	**Mean ± standard deviation**					**KW test**	**Mann-Whitney U tests**			
**TD**	meanNN	939 ± 129	925 ± 138	923 ± 134	904 ± 123	906 ± 123	NS	NS	NS	NS	NS
	sdNN	50.0 ± 20.9	44.6 ± 16.8	36.8 ± 14.6	32.8 ± 14.7	29.6 ± 13.2	****	*	**	*	NS
	cvNN	0.05 ± 0.02	0.05 ± 0.02	0.04 ± 0.01	0.04 ± 0.01	0.03 ± 0.01	****	*	**	*	NS
	sdaNN1	18.9 ± 13.0	18.2 ± 10.4	16.4 ± 9.5	14.8 ± 9.5	13.7 ± 8.9	**	NS	NS	NS	NS
	rmssd	39.7 ± 19.9	32.0 ± 16.5	23.0 ± 10.9	19.9 ± 11.1	19.1 ± 10.7	****	**	**	*	NS
	pNN50	0.20 ± 0.17	0.13 ± 0.15	0.06 ± 0.08	0.04 ± 0.07	0.04 ± 0.07	****	**	****	*	NS
	pNNl20	0.45 ± 0.20	0.54 ± 0.21	0.67 ± 0.20	0.75 ± 0.19	0.78 ± 0.18	****	**	****	**	NS
	renyi4	4.00 ± 0.53	3.86 ± 0.52	3.56 ± 0.56	3.37 ± 0.57	3.22 ± 0.55	****	*	**	*	NS
	shannon_h	4.45 ± 0.52	4.30 ± 0.51	4.02 ± 0.55	3.83 ± 0.56	3.68 ± 0.54	****	*	**	*	NS
**FD**	LF	242 ± 325	191 ± 206	113 ± 141	80 ± 103	70 ± 112	****	*	**	**	NS
	HF	133 ± 174	89 ± 118	41 ± 49	29 ± 38	22 ± 29	****	**	****	*	NS
	P	625 ± 660	500 ± 407	348 ± 345	275 ± 290	241 ± 300	****	NS	**	*	NS
	LF/HF	2.79 ± 3.20	3.62 ± 3.73	4.10 ± 3.48	4.17 ± 3.60	4.77 ± 5.34	***	*	*	NS	NS
	LF/P	0.38 ± 0.16	0.38 ± 0.16	0.34 ± 0.15	0.30 ± 0.14	0.29 ± 0.16	**	NS	NS	NS	NS
	HF/P	0.22 ± 0.13	0.18 ± 0.13	0.13 ± 0.09	0.12 ± 0.11	0.12 ± 0.11	****	**	**	NS	NS
	LFn	0.64 ± 0.17	0.68 ± 0.18	0.72 ± 0.16	0.73 ± 0.16	0.71 ± 0.19	***	*	*	NS	NS
	HFn	0.36 ± 0.17	0.32 ± 0.18	0.28 ± 0.16	0.27 ± 0.16	0.29 ± 0.19	***	*	*	NS	NS

Abbreviations: MA, method of analysis; TD, time domain; FD, frequency domain; N: number of subjects; KW test: Kruskal-Wallis test; level of significance:

****p<10^−20^,

***p<10^−10^,

**p<Bonferroni criterion = 0.0007,

*p<0.01, NS, no significance.

**Table 8:** Descriptive statistics and results of Kruskal-Wallis test followed by Mann-Whitney U tests with respect to nonlinear HRV indices comparing each two age decades in males (Tests II, comparison of five different age decades: 1 = 25–34, 2 = 35–44, 3 = 45–54, 4 = 55–64 and 5 = 65–74 years).

		**1 (N = 330)**	**2 (N = 292)**	**3 (N = 235)**	**4 (N = 183)**	**5 (N = 84)**	**1–5**	**1 vs. 2**	**2 vs. 3**	**3 vs. 4**	**4 vs. 5**
MA	HRV-index	Mean ± standard deviation					KW test	Mann-Whitney U tests			
**SD**	shannon_SD	3.15 ± 0.42	2.95 ± 0.44	2.62 ± 0.47	2.42 ± 0.49	2.29 ± 0.47	****	**	****	**	NS
	forbword	25.5 ± 9.5	30.1 ± 9.3	36.2 ± 8.5	38.9 ± 8.6	39.8 ± 9.1	****	**	****	*	NS
	wpsum02	0.45 ± 0.22	0.53 ± 0.22	0.67 ± 0.20	0.73 ± 0.20	0.79 ± 0.18	****	**	**	*	NS
	wpsum13	0.10 ± 0.09	0.10 ± 0.09	0.08 ± 0.08	0.07 ± 0.08	0.05 ± 0.07	***	NS	*	*	NS
	wsdvar	1.32 ± 0.42	1.23 ± 0.43	1.02 ± 0.46	0.88 ± 0.48	0.75 ± 0.43	****	*	**	*	*
	phvar5	0.48 ± 0.19	0.39 ± 0.19	0.28 ± 0.17	0.22 ± 0.16	0.19 ± 0.16	****	**	**	*	NS
	plvar20	0.07 ± 0.16	0.12 ± 0.22	0.25 ± 0.28	0.36 ± 0.32	0.43 ± 0.31	****	**	****	**	NS
	fwrenyi025	3.49 ± 0.31	3.33 ± 0.33	3.09 ± 0.37	2.95 ± 0.40	2.89 ± 0.39	****	**	****	*	NS
	fwrenyi4	2.55 ± 0.54	2.31 ± 0.54	1.93 ± 0.51	1.72 ± 0.48	1.59 ± 0.46	****	**	****	**	NS
**DFA**	α1	0.93 ± 0.20	0.99 ± 0.23	1.10 ± 0.22	1.15 ± 0.22	1.13 ± 0.26	****	**	**	NS	NS
	α2	0.85 ± 0.23	0.87 ± 0.21	0.96 ± 0.21	0.96 ± 0.19	1.01 ± 0.21	***	NS	**	NS	NS
**CE**	H_c_^3,3^	0.80 ± 0.08	0.77 ± 0.08	0.71 ± 0.09	0.67 ± 0.09	0.65 ± 0.08	****	**	****	*	NS
**STSD**	ST_MP	0.70 ± 0.07	0.72 ± 0.07	0.75 ± 0.06	0.76 ± 0.07	0.75 ± 0.08	***	**	**	NS	NS
	ST_0V	0.06 ± 0.08	0.04 ± 0.06	0.03 ± 0.06	0.02 ± 0.05	0.03 ± 0.07	***	**	*	NS	NS
	ST_1V	0.49 ± 0.12	0.52 ± 0.13	0.55 ± 0.14	0.57 ± 0.14	0.55 ± 0.17	**	*	NS	NS	NS
	ST_2V	0.26 ± 0.10	0.25 ± 0.10	0.20 ± 0.10	0.19 ± 0.11	0.19 ± 0.12	***	NS	**	NS	NS
	ST_INC	0.09 ± 0.05	0.10 ± 0.05	0.07 ± 0.04	0.07 ± 0.05	0.06 ± 0.04	***	NS	**	NS	NS
	ST_DESC	0.08 ± 0.04	0.08 ± 0.05	0.07 ± 0.04	0.06 ± 0.04	0.06 ± 0.05	**	NS	**	NS	NS
	ST_PEAK	0.14 ± 0.04	0.13 ± 0.05	0.14 ± 0.06	0.14 ± 0.06	0.15 ± 0.06	NS	NS	NS	NS	NS
	ST_VAL	0.14 ± 0.05	0.13 ± 0.05	0.14 ± 0.06	0.14 ± 0.05	0.16 ± 0.06	**	*	NS	NS	NS
	ST_2LV	0.17 ± 0.07	0.18 ± 0.08	0.14 ± 0.07	0.13 ± 0.08	0.12 ± 0.08	***	NS	**	NS	NS
	ST_2UV	0.28 ± 0.08	0.26 ± 0.09	0.28 ± 0.09	0.29 ± 0.09	0.31 ± 0.11	*	*	NS	NS	NS
**PPA**	SD1	28.1 ± 14.1	22.7 ± 11.7	16.3 ± 7.7	14.0 ± 7.8	13.4 ± 7.6	****	**	**	*	NS
	SD2	64.4 ± 27.0	58.3 ± 21.7	49.1 ± 20.0	43.8 ± 19.8	39.1 ± 18.1	****	*	**	*	NS
	SD1/SD2	0.44 ± 0.12	0.39 ± 0.13	0.34 ± 0.13	0.32 ± 0.11	0.36 ± 0.19	****	**	**	NS	NS
**SPPA**	SPPA_c_4	2.03 ± 1.19	2.02 ± 1.25	2.21 ± 1.22	2.27 ± 1.40	2.46 ± 1.41	NS	NS	NS	NS	NS
	SPPA_c_5	12.5 ± 3.2	12.6 ± 3.3	12.1 ± 3.0	12.0 ± 3.6	11.8 ± 3.5	NS	NS	NS	NS	NS
	SPPA_c_6	34.8 ± 5.1	34.4 ± 5.3	34.2 ± 5.0	33.8 ± 5.2	33.4 ± 5.9	NS	NS	NS	NS	NS
	SPPA_c_7	35.0 ± 5.6	35.4 ± 5.5	35.8 ± 5.7	36.6 ± 6.1	36.9 ± 7.5	NS	NS	NS	NS	NS
	SPPA_c_8	13.2 ± 2.4	13.1 ± 2.6	13.3 ± 2.6	13.1 ± 2.7	13.3 ± 3.1	NS	NS	NS	NS	NS
	SPPA_c_9	1.94 ± 1.10	1.90 ± 1.15	1.75 ± 1.25	1.63 ± 1.21	1.47 ± 1.15	**	NS	NS	NS	NS
	SPPA_r_4	2.11 ± 0.98	2.05 ± 0.96	1.95 ± 0.96	1.90 ± 0.86	1.80 ± 0.91	NS	NS	NS	NS	NS
	SPPA_r_5	12.7 ± 2.8	12.5 ± 2.8	12.3 ± 3.0	11.9 ± 3.0	11.0 ± 4.4	*	NS	NS	NS	NS
	SPPA_r_6	34.2 ± 4.4	34.8 ± 4.2	35.5 ± 4.0	35.9 ± 4.2	36.7 ± 4.8	**	NS	NS	NS	NS
	SPPA_r_7	34.5 ± 5.5	34.7 ± 5.1	34.7 ± 4.8	35.3 ± 4.6	35.9 ± 5.1	NS	NS	NS	NS	NS
	SPPA_r_8	14.4 ± 3.1	13.8 ± 3.1	13.2 ± 3.2	12.5 ± 3.1	11.7 ± 4.0	***	NS	NS	NS	NS
	SPPA_r_9	1.43 ± 0.77	1.59 ± 0.88	1.71 ± 0.75	1.79 ± 0.84	1.84 ± 0.97	**	NS	*	NS	NS
	SPPA_entropy	4.02 ± 0.12	4.01 ± 0.10	4.01 ± 0.13	3.99 ± 0.14	3.94 ± 0.22	*	NS	NS	NS	NS
**IA**	AS1	51.1 ± 3.9	50.7 ± 3.7	50.0 ± 3.0	50.1 ± 2.9	50.2 ± 3	*	NS	NS	NS	NS
	AS2	54.2 ± 6.2	53.5 ± 6.3	51.9 ± 6.1	52.9 ± 7.1	54.5 ± 7.8	**	NS	*	NS	NS
	AS3	0.03 ± 0.06	0.03 ± 0.06	0.03 ± 0.08	0.04 ± 0.11	0.06 ± 0.11	*	NS	*	NS	NS
**ACOR and AMI**	a21rr	−2.35 ± 0.29	−2.24 ± 0.31	−2.10 ± 0.32	−2.05 ± 0.33	−2.05 ± 0.37	****	**	**	NS	NS
	a31rr	−1.31 ± 0.12	−1.28 ± 0.12	−1.21 ± 0.13	−1.18 ± 0.15	−1.16 ± 0.17	****	**	**	NS	NS
	x2peakrr	5.79 ± 1.89	6.35 ± 2.16	7.23 ± 2.51	7.79 ± 2.94	7.35 ± 2.89	***	**	**	NS	NS
	y2peakrr	0.42 ± 0.13	0.42 ± 0.12	0.45 ± 0.16	0.45 ± 0.17	0.49 ± 0.19	NS	NS	NS	NS	NS
	amax21rr	−0.50 ± 0.14	−0.46 ± 0.14	−0.40 ± 0.14	−0.37 ± 0.13	−0.39 ± 0.15	****	**	**	NS	NS
	a21rrcor	−0.33 ± 0.15	−0.27 ± 0.15	−0.22 ± 0.15	−0.21 ± 0.12	−0.25 ± 0.21	****	**	**	NS	NS
	a31rrcor	−0.32 ± 0.11	−0.28 ± 0.12	−0.21 ± 0.10	−0.19 ± 0.09	−0.19 ± 0.09	****	**	**	NS	NS
	x2peakrrcor	6.80 ± 3.32	7.81 ± 3.81	8.90 ± 4.67	10.30 ± 5.90	9.94 ± 6.37	**	*	NS	NS	NS
	y2peakrrcor	0.35 ± 0.16	0.35 ± 0.16	0.34 ± 0.19	0.31 ± 0.23	0.32 ± 0.24	NS	NS	NS	NS	NS
	amax21rrcor	−0.12 ± 0.05	−0.10 ± 0.05	−0.09 ± 0.05	−0.09 ± 0.04	−0.09 ± 0.06	***	*	*	NS	NS

Abbreviations: MA, method of analysis; SD, symbolic dynamic; DFA, detrended fluctuation analysis; CE, compression entropy; STSD, short-term symbolic dynamic; PPA, Poincaré plot analysis; SPPA, segmented Poincaré plot analysis; IA, irreversibility analysis; ACOR and AMI, auto-correlation and auto-mutual information; N: number of subjects; KW test: Kruskal-Wallis test; level of significance:

****p<10–20,

***p<10–10,

**p<Bonferroni criterion = 0.0007,

*p<0.01, NS, no significance.

**Table 9:** Results of Mann-Whitney U tests of linear HRV indices comparing five age matched female and male groups (Tests III, comparison of gender differences in five different age decades).

		**Females vs. Males**				
**MA**	**HRV-index**	**25–34 years**	**35–44 years**	**45–54 years**	**55–64 years**	**65–74 years**
**TD**	meanNN	**	NS	NS	NS	NS
	sdNN	NS	NS	NS	NS	NS
	cvNN	NS	NS	NS	NS	NS
	sdaNN1	NS	NS	NS	NS	NS
	rmssd	NS	NS	NS	NS	NS
	pNN50	NS	NS	NS	NS	NS
	pNNl20	NS	NS	NS	NS	NS
	renyi4	NS	NS	NS	NS	NS
	shannon_h	NS	NS	NS	NS	NS
**FD**	LF	*	*	NS	NS	NS
	HF	NS	*	**	NS	NS
	P	NS	NS	NS	NS	NS
	LF/HF	**	**	**	**	*
	LF/P	**	**	*	NS	NS
	HF/P	**	**	**	**	*
	LFn	**	**	**	**	*
	HFn	**	**	**	**	*

Abbreviations: level of significance:

****p<10^−20^,

***p<10^−10^,

**p<Bonferroni criterion = 0.0007,

*p<0.01, NS, no significance; MA, method of analysis; TD, time domain; FD, frequency domain.

**Table 10:** Results of Mann-Whitney U tests of nonlinear HRV indices comparing five age matched female and male groups (Tests III, comparison of gender differences in five different age decades).

		**Females vs. Males**				
**MA**	**HRV-index**	**25–34 years**	**35–44 years**	**45–54 years**	**55–64 years**	**65–74 years**
**SD**	shannon_SD	*	*	NS	NS	NS
	forbword	*	*	NS	NS	NS
	wpsum02	NS	NS	NS	NS	NS
	wpsum13	NS	NS	NS	NS	NS
	wsdvar	NS	NS	NS	NS	NS
	phvar5	NS	NS	NS	NS	NS
	plvar20	NS	*	*	NS	NS
	fwrenyi025	*	*	NS	NS	NS
	fwrenyi4	*	*	*	NS	NS
**DFA**	α1	*	*	*	*	NS
	α2	NS	*	NS	NS	NS
**CE**	H_c_^3,3^	NS	NS	NS	NS	NS
**STSD**	ST_MP	*	**	*	NS	NS
	ST_0V	*	**	*	*	NS
	ST_1V	**	**	*	NS	NS
	ST_2V	*	*	NS	NS	NS
	ST_INC	NS	NS	NS	NS	NS
	ST_DESC	NS	NS	NS	NS	NS
	ST_PEAK	NS	NS	NS	NS	NS
	ST_VAL	**	**	*	NS	NS
	ST_2LV	NS	NS	NS	NS	NS
	ST_2UV	**	**	NS	NS	NS
**PPA**	SD1	NS	NS	NS	NS	NS
	SD2	NS	NS	NS	NS	NS
	SD1/SD2	*	*	*	*	NS
**SPPA**	SPPA_c_4	NS	NS	NS	NS	NS
	SPPA_c_5	NS	NS	NS	NS	NS
	SPPA_c_6	NS	NS	NS	NS	NS
	SPPA_c_7	NS	NS	NS	NS	NS
	SPPA_c_8	NS	NS	NS	NS	NS
	SPPA_c_9	NS	NS	NS	NS	NS
	SPPA_r_4	**	*	NS	NS	NS
	SPPA_r_5	**	*	*	NS	NS
	SPPA_r_6	*	NS	NS	NS	NS
	SPPA_r_7	****	**	*	NS	NS
	SPPA_r_8	**	**	**	NS	NS
	SPPA_r_9	NS	NS	NS	NS	NS
	SPPA_entropy	NS	NS	NS	NS	NS
**IA**	AS1	**	**	NS	NS	NS
	AS2	**	**	NS	NS	NS
	AS3	**	**	NS	NS	NS
**ACOR and AMI**	a21rr	*	**	**	*	NS
	a31rr	*	*	**	NS	NS
	x2peakrr	*	*	*	*	NS
	y2peakrr	**	*	NS	NS	NS
	amax21rr	*	*	*	*	NS
	a21rrcor	*	*	*	*	NS
	a31rrcor	*	*	*	NS	NS
	x2peakrrcor	**	**	**	*	NS
	y2peakrrcor	**	**	*	*	NS
	amax21rrcor	*	**	*	NS	NS

Abbreviations: level of significance:

****p<10^−20^,

***p<10^−10^,

**p<Bonferroni criterion = 0.0007,

*p<0.01, NS, no significance; MA, method of analysis; SD, symbolic dynamic; DFA, detrended fluctuation analysis; CE, compression entropy; STSD, short-term symbolic dynamic; PPA, Poincaré plot analysis; SPPA, segmented Poincaré plot analysis; IA, irreversibility analysis; ACOR and AMI, auto-correlation and auto-mutual information.

### 1 Time Domain (TD)

**Fig 1:**
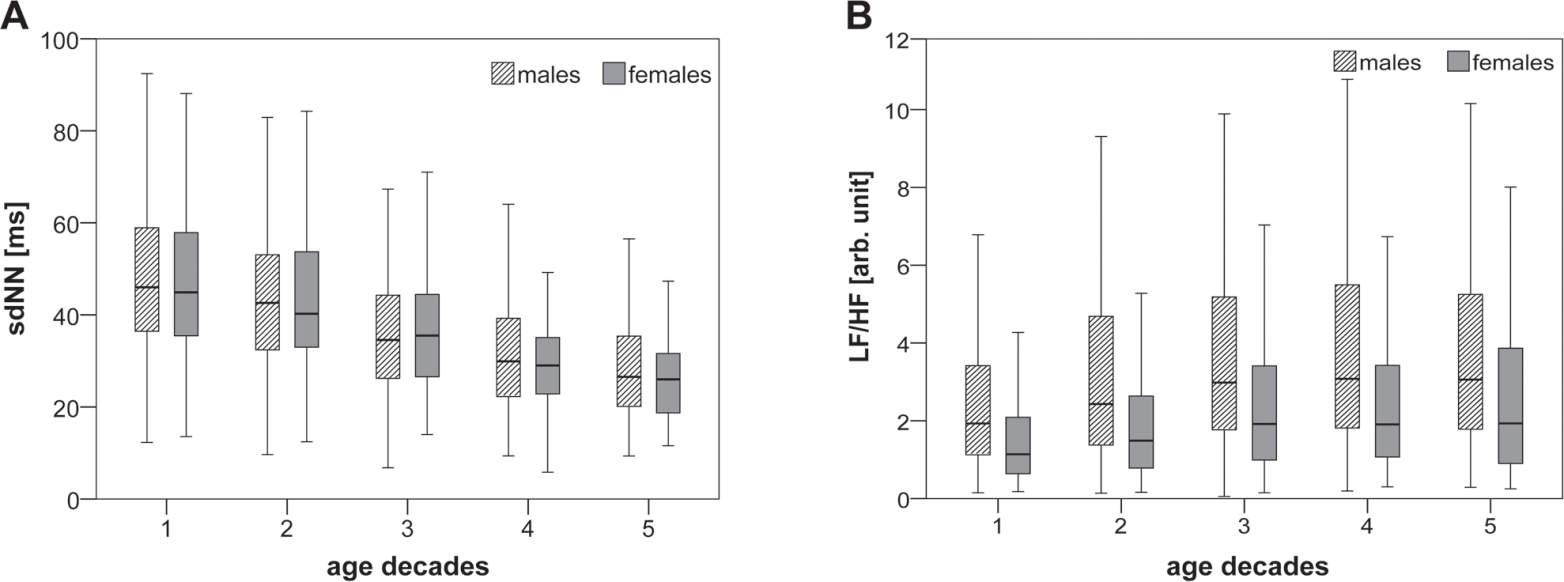
Box plots for sdNN and LF/HF for five investigated age decades divided by gender. The box plots show two selected HRV indices (A: sdNN (TD) and B: LF/HF (FD)) for the five investigated age decades (1: 25–34, 2: 35–44, 3: 45–54, 4: 55–64 and 5: 65–74 years) and divided into males (black cross-striped boxes) and females (white boxes). The boxes show the data between the 25th and 75 Percentile, the middle line represents the median.

For HRV indices from TD, Test I revealed considerable differences comparing the age groups 25–49 years and 50–74 years, separately by gender (YF vs. EF and YM vs. EM). Furthermore, the significance levels of the TD indices obtained for the test YF vs. EF were comparable to those achieved for the test YM vs. EM. Most of the investigated TD indices (sdNN, cvNN, rmssd, pNN50, pNNl20, renyi4 and shannon_h) showed the highest levels of significance (****). The index sdaNN1 showed a highly significant (***) difference, however, the meanNN revealed no significant difference. Diminished median values of sdNN (Fig. 1A), cvNN, sdaNN1, rmssd, pNN50, renyi4 and shannon_h and an increased pNNl20 index were associated with ageing, irrespectively of gender. The comparison of genders (YF vs. YM and EF vs. EM) considering two age ranges offered a slight significance (at least *) of the TD index meanNN. The median value of meanNN was higher in male groups than in female groups. These findings, as expected, reveal a considerably diminished HRV in the older age groups regardless of female or male gender. Furthermore, the influence of gender on TD indices of HRV analysis is more marginal than initially expected.

The more extensive results of Test II demonstrated, irrespective of the gender: (a) a general HRV decline (at least **) over all ages (see Kruskal-Wallis test on the age groups 1–5) confirming the results of Test I; (b) highest significant changes comparing the age groups 2 and 3 (age range 35–54 years); and (c) no significant differences at all comparing the age groups 4 and 5 (age range 55–74 years). Additionally, with regard to age, (d) significant gender differences for all (with exception of meanNN) of the TD indices were not present.

### 2 Frequency Domain (FD)

According to Test I, all indices from FD differed considerably (at least **) between the young male group and the elderly male group (YM vs. EM). When comparing FD indices of the two different female age groups (YF vs. EF), the indices LF, HF, P, and HF/P showed significant differences (at least **). For both genders, the values of the indices LF, HF, P, LF/P, HF/P and HFn diminished with ageing, whereas LF/HF (Fig. 1B) and LFn increased with higher age. The comparison of both gender young age groups (YF vs. YM) revealed significant values (at least **) for the indices LF, LF/HF, LF/P, HF/P, LFn and HFn. When comparing between elderly females and males (EF vs. EM), these indices were also significant, but not as much (at least *). The median of the indices LF, LF/HF, LF/P and LFn increased and the median of the indices HF/P and HFn decreased in the male groups as compared to the age-related female groups.

The more extensive results of Test II demonstrated, irrespective of gender: (a) a general power decline in the frequency bands; (b) a slight (*) decrease of vagal activity and increase of the sympathetic influence with growing age when comparing the age decades 25–34 and 35–44 years (and also in males when comparing the age decades 35–44 years and 45–54 years); and (c) no significant changes of normalized frequency indices between the age groups 3 to 5 (age range 45–74 years). Additionally, with regard to all age decades, namely 1–5 (age range 25–74 years) for both genders, (d) there were significant (*) gender differences for the normalized FD indices and for the LF/HF ratio which describe the balance between the two arms of the cardiac autonomic control system. The median value of LH/HF was significantly increased in each male group compared to the age-related female group indicating an increased sympathetic activity and decreased vagal activity in male subjects. When comparing the gender groups with ages 65–74 years, the differences (**) in the frequency indices became smaller (*).

In this study, we also calculated the indices of frequency domain by using the parametric spectral analysis with a window length of 1024, a Blackman-Harris window, a fixed order of 16 [60], and the Burg algorithm. Comparing the results of the fast Fourier transform and of the parametric spectral analysis, the obtained frequency domain indices achieved differing values but similar significances. For this reason, frequency domain indices and associated results attained using the parametric spectral analysis are not shown here.

### 3 Nonlinear Dynamics

Assessing the results of Test I (comparing the age groups 25–49 years and 50–74 years), all traditional SD indices reached the highest significant level (****), irrespective of gender (YF vs. EF and YM vs. EM). For both genders, the values of the SD indices shannon_SD, wpsum13, wsdvar, phvar5, fwrenyi025 and fwrenyi4 decreased with increasing age, whereas the indices forbword, wpsum02 and plvar20 increased with increasing age. When comparing genders (YF vs. YM and EF vs. EM) considering two age ranges, there was a significant (**) decrease of the index fwrenyi4 in young males as compared to young females.

**Fig 2:**
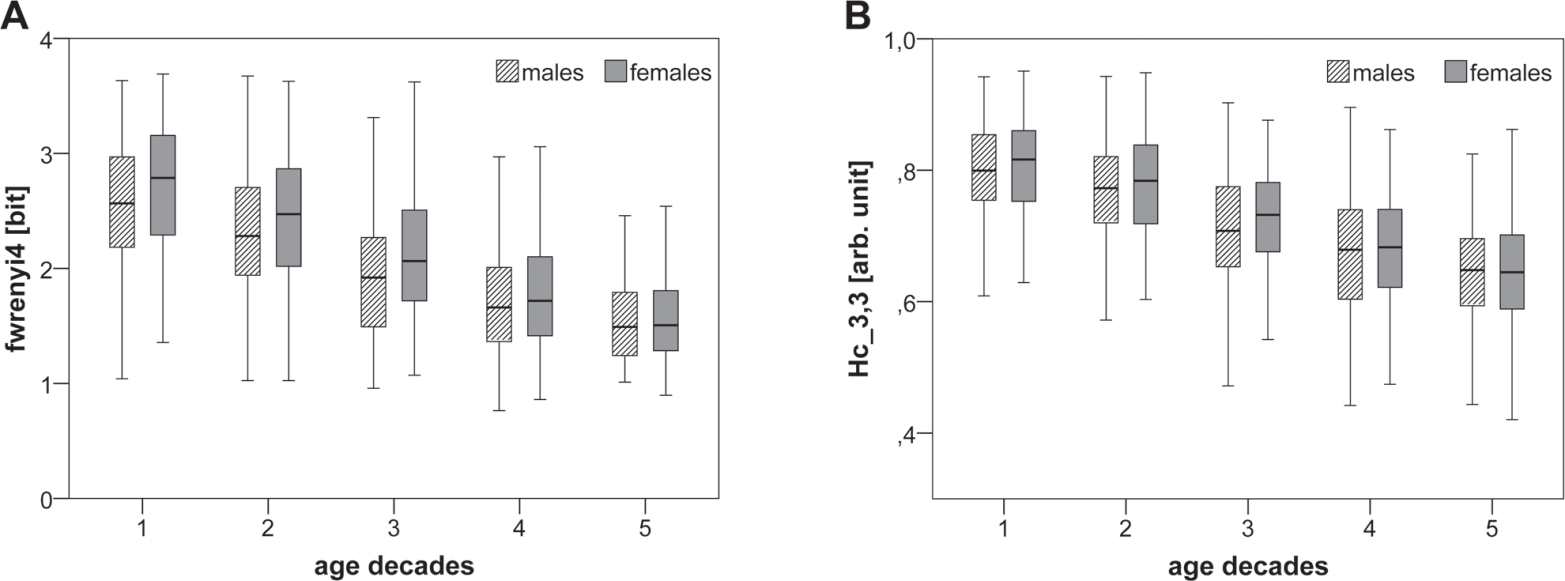
Box plots for fwrenyi4 and H_c_^3,3^ for five investigated age decades divided by gender. The box plots show two selected HRV indices (A: fwrenyi4 (SD) and B: H_c_^3,3^ (CE)) for the five investigated age decades (1: 25–34, 2: 35–44, 3: 45–54, 4: 55–64 and 5: 65–74 years) and divided into males (black cross-striped boxes) and females (white boxes). The boxes show the data between the 25th and 75 Percentile, the middle line represents the median.

The more extensive results of Test II demonstrated, irrespective of gender: (a) a general decline in both complexity (for example see fwrenyi4 in Fig. 2A) and variability throughout all age groups; (b) a decrease in complexity which is most pronounced when comparing males between 35–44 years and 45–54 years; and (c) no meaningful changes of traditional SD indices between age groups 4 and 5 (age range 55 to 74 years). Additionally, with regard to the age decades, (d) meaningful gender differences (≥**) for any of the SD indices were not existent.

From DFA, the indices α1 and α2 showed a marked increase in significance (at least **) with increased age in Test I, irrespective of the gender (YF vs. EF and YM vs. EM). Only slight differences in the indices were apparent when comparing the age-related gender groups (YF vs. YM and EF vs. EM).

The more extensive results of Test II demonstrated, irrespective of gender: (a) a general increase of short- and long-term fractal correlation throughout all age groups (Kruskal-Wallis test); and (b) a significant increase (at least *) of the index α1 only between the age groups 1 to 3 (age range 25–54 years). Furthermore, (c) the index α2 was only significantly different (**) between males aged 35–44 years and 45–54 years. Additionally (d), with regard to age decades, meaningful gender differences (≥**) for all of the DFA indices were not existent.

Irrespective of the gender (YF vs. EF and YM vs. EM), the index H_c_^3,3^ from CE revealed the highest significant (****) decrease (Fig. 2B) with increased age in Test I. The use of another window length and look-ahead buffer length for CE calculation resulted in a lower but also highest significant decrease with aging compared to the index H_c_^3,3^ (b = 3 and w = 3). Thus, only the index H_c_^3,3^ is presented in this study. Comparing the CE index of the age-related gender groups (YF vs. YM and EF vs. EM) revealed no differences.

The more extensive results of Test II demonstrated, irrespective of gender: (a) a general decrease of H_c_^3,3^ with ageing as sign of diminishing information within the signal, thus confirming Test I; (b) a decrease of H_c_^3,3^ which is most pronounced (****) when comparing males between 35–44 years and 45–54 years; and (c) no meaningful changes (≥**) in the CE index between age groups 3 to 5 (age range 45 to 74 years). With regard to age decades, (d) no gender differences within index H_c_^3,3^ were found.

Almost all indices of STSD revealed considerable differences (at least **) when comparing the young (25–49 years) and the elderly (50–74 years) age groups separated by gender (Test I, YF vs. EF and YM vs. EM). ST_PEAK and ST_VAL for both genders and ST_2UV considering females were the only indices that were changed only in trend (*) or statistically not significant. For both genders, the values of the STSD indices ST_0V, ST_2V, ST_INC, ST_DESC and ST_2LV diminished with increasing age. However, the two indices ST_MP and ST_1V rose with increasing age. The comparison of the two gender-related young age groups (YF vs. YM) revealed significant values (at least **) for the indices ST_MP, ST_0V, ST_1V, ST_2V, ST_VAL and ST_2UV. The median of the indices ST_MP and ST_1V increased and the median of the indices ST_0V, ST_2V, ST_VAL and ST_2UV decreased in the young male group as compared to the young female group. None of the STSD indices showed significant changes when comparing elderly females and elderly males (EF vs. EM).

The more extensive results of Test II demonstrated (without the above mentioned indices ST_PEAK, ST_VAL and ST_2UV): (a) a general decline in both complexity and variability throughout all age groups for both genders; (b) ST_MP and ST_0V in the male group as the only STSD indices that could significantly differentiate (**) between the age groups 1 and 2 (25 to 34 years and 35 to 44 years); and (c) that the highest number of significant indices (ST_MP and ST_2V significant in both gender groups and ST_INC, ST_DESC and ST_2LV only in the male groups) was obtained when comparing the age groups 2 and 3 (35 to 54 years). Additionally (d), the age decades 1–2 (age range 25–44 years) revealed significant (**) gender differences for the STSD indices ST_1V (increased in males) and ST_VAL and ST_2UV (both decreased in the male groups). Comparing the gender groups aged 45–74 years revealed a disappearance in variation for the STSD indices.

Assessing the results of Test I, all traditional PPA indices (SD1, SD2 and SD1/SD2) reached the highest significant level (****) differentiating between young and elderly subject groups, irrespective of gender (YF vs. EF and YM vs. EM). Decreased PPA indices in elderly subjects compared to young subjects reflecting a decreased HRV linked to the ageing. Comparing young females and elderly females, only the SPPA indices SPPA_r_7 and SPPA_r_8 showed highest significance (****) differences. Comparing the young and the elderly male groups revealed at least significant changes (**) of the indices SPPA_r_6, SPPA_r_8 and SPPA_r_9. The comparison of the two gender-related young age groups (YF vs. YM) revealed significant values (at least **) for the PPA index SD1/SD2 (decreased in male subjects) and for the SPPA indices SPPA_r_4 to SPPA_r_8. No meaningful differences (≥**) between elderly females and males (EF vs. EM) could be determined.

**Fig 3:**
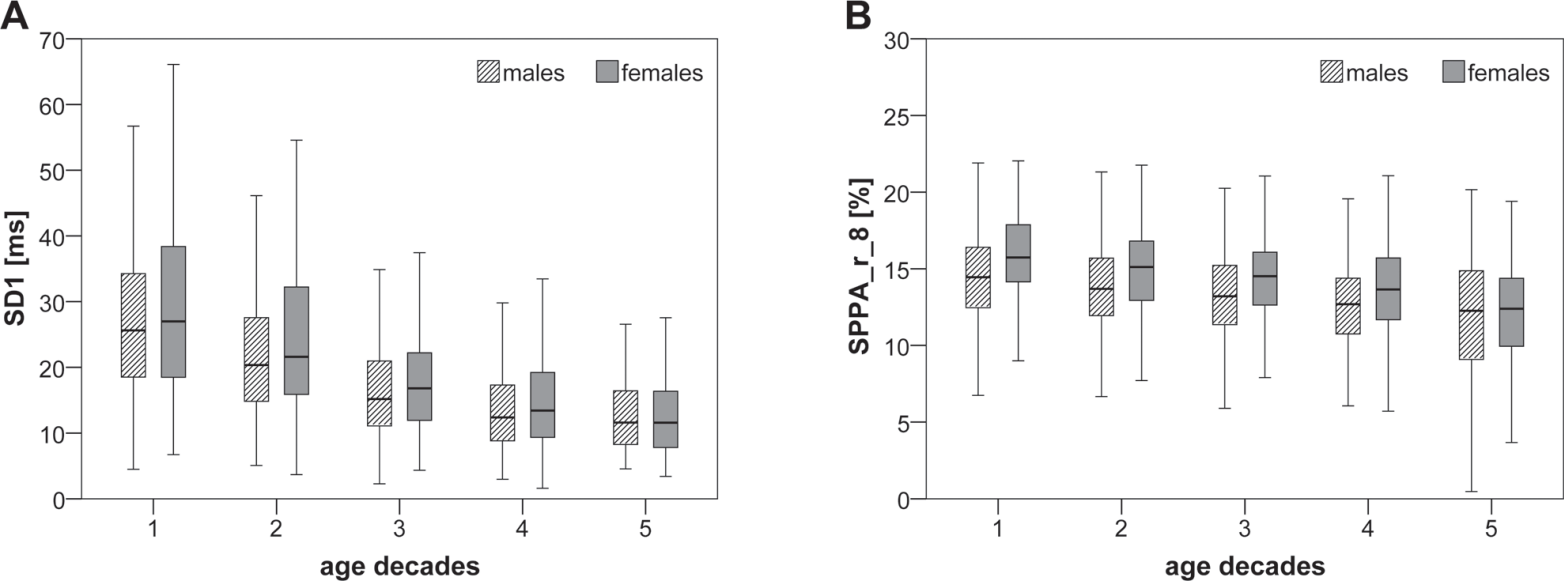
Box plots for SD1 and SPPA_r_8 for five investigated age decades divided by gender. The box plots show two selected HRV indices (A: SD1 (PPA) and B: SPPA_r_8 (SPPA)) for the five investigated age decades (1: 25–34, 2: 35–44, 3: 45–54, 4: 55–64 and 5: 65–74 years) and divided into males (black cross-striped boxes) and females (white boxes). The boxes show the data between the 25th and 75 Percentile, the middle line represents the median.

The more extensive results of Test II demonstrated: (a) a perceptible decline of all PPA indices, occurring only between the age groups 1 to 4 (age range 25 to 64 years) and more pronounced between the male groups, suggests a decrease of the HRV (for an example see SD1 in Fig. 3A), independent of gender; and (b) no further significant changes of PPA indices when comparing the age groups 4 to 5 (55–74 years). Furthermore (c), the SPPA indices SPPA_c_7, SPPA_r_5, SPPA_r_7 and SPPA_r_8 (Fig. 3B) in females and SPPA_c_9, SPPA_r_6, SPPA_r_8 and SPPA_r_9 in males showed a general age-dependent influence (at least ** using the Kruskal-Wallis test). This age-dependent influence on SPPA indices could not be found when differentiating between successive age decades using the Mann-Whitney U test. Further, (d) with regard to age decades there were no meaningful gender differences on the PPA indices. However significant (at least **) gender differences on the SPPA indices SPPA_r_4 and SPPA_r_5 in the age range 25–34 years and on the indices SPPA_r_7 and SPPA_r_8 in the age range 25–54 years were proven. In the last age decades, the gender differences disappeared for the mentioned SPPA indices.

From IA, the indices AS1 and AS3 revealed slightly significant (at least *) age-dependent differences in Test I, irrespective of the gender (YF vs. EF and YM vs. EM). Whereas all IA indices revealed significantly increased (at least **) values in young males (a sign of higher asymmetry in NN interval time series) in comparison to females (YF vs. YM), no significances were observed between the elderly groups of subjects (EF vs. EM).

The more extensive results of Test II revealed: (a) for the indices AS1 in both genders and AS2 and AS3 only in males a low degree of significant (*) age-dependent differences (Kruskal-Wallis test); and (b) for all IA indices significant (**) gender differences in the age decades 1–2 (age range 25–44 years). Additionally (c), a comparison of gender groups aged 45–74 years revealed a disappearance in these gender differences.

From AMI and ACOR, all indices with exception of y2peakrrcor (ACOR index, *) revealed considerable (at least **) age-dependent differences, independent of the gender (YF vs. EF and YM vs. EM) in Test I. Increasing decay indices from the AMI and ACOR analysis (steep declines of the curve progressions) with age, reflect an increasing predictability and loss of complexity. With the exception of the ACOR index a31rrcor, for all ACOR and AMI indices (aside from y2peakrr and y2peakrrcor where all indices were increased in male subject groups) significant values (at least **) were revealed when comparing the two gender-related young age groups (YF vs. YM). Only the ACOR index x2peakrrcor was significantly higher in the elderly male group as compared to the elderly female group (EF vs. EM).

The more extensive results of Test II revealed: (a) significantly increasing decay indices from the AMI and ACOR analysis comparing the age groups 1 and 2 as well as 2 and 3 (age range 25–54 years) in particular in males (**) but also at least in trend in females (*); and (b) no significant age-dependent differences comparing the AMI and ACOR indices of the age groups 3 and 4 as well as 4 and 5 (age range 45–74 years) for both genders. Additionally (c), with regard to the age decades 1–3 (age range 25–54 years), there are significant (at least **) gender differences on the AMI indices a21rr and a31rr and on the ACOR indices y2peakrr, x2peakrrcor, y2peakrrcor and amax21rrcor. Finally (c), when comparing the results of ACOR and AMI for both gender groups aged 55–74 years, no meaningful gender differences could be found.

## Discussion

In accordance with our previous study [39], linear and nonlinear indices of short-term HRV analysis were calculated from NN interval time series of a representative group of healthy subjects. The enrolled subjects represent a random sample of the average population (KORA S4 database). Based on the previous study [39], in this subsequent study, besides the age dependency we focused especially on the general gender dependency (young vs. elderly subjects) on HRV indices and on the gender-dependent HRV development in five age decades.

Generally, independent of gender, the HRV and complexity were diminished and the predictability was higher in the elderly subject group (age 50–74 years) compared to the younger subject group (25–49 years), as seen in Test I. Test II affirms this findings, as clearly illustrated in Figs. 1–3, whereas significant modifications of the HRV indices in terms of age disappeared within the last two age decades (age range 55–74 years). Furthermore, general dependence on gender for many HRV indices, particularly from FD, STSD, SPPA, IA, ACOR and AMI (highly significant), was proven in young subjects (in Test I, there were 39 significant indices when comparing YF and YM). These dependencies disappear with increasing age (as the results in Test II show, where only marginal significances for FD indices were found in the age decade 65–74 years). According to HRV analysis methods, the influences of age and gender on HRV indices differed partly, whereas in general the gender influences were considerably weaker than the age influences.

An individual consideration of the several method domains can lead to following conclusions:

### 1 Time Domain

Previous research [22, 26, 30, 31, 33] suggests that the time domain indices sdNN, cvNN, sdaNN1, rmssd and pNN50 which quantify the variability of a NN interval time series are inversely related to age for both genders. This effect was also present as a trend in our study. Further on, Beckers et al. [33] showed a stabilization of the decline in linear indices around the age category of 40 years confirming our findings that differences in the time domain indices converge at the age >45 years and disappear at the age >55 years in both genders.

Studies of Bigger et al. [28] and Corrales et al. [61] revealed only small insignificant differences in NN interval variability between females and males in contrast to other studies [29, 30, 32, 62, 63] showing a significantly lower SDNN for females than for males. In this study, no significances (only a trend toward lower SDNN levels in females when comparing median values between females and males) in SDNN were revealed. The reason could be due to the application of a lowest significance level of p<0.01 instead of p<0.05 [29, 30, 32, 62], a different length of time series, here: short-term 5-min, whereas [29, 30, 32]: used long-term 24-h and a non-representative number of healthy subjects [62]: 67, and [32]: 33 healthy subjects. Furthermore, Greiser et al. [24] showed an overall inverse association of age with SDNN in women (45–83 years), while in men SDNN decreased between the ages 45 and 74 years, followed by increased SDNN values in the older male age groups (SDNN across 10-year age groups 32.1, 26.9, 27.1 and 24.8 ms in women, 29.3, 25.9, 23.8 and 25.7 ms in men). In this study, we can confirm such behavior across 10-year age groups (ranging from 25–74 years). Subjects aged over 74 years where not enrolled in this study. Thus, the described increase of SDNN in males over 74 years [24] could not be proven.

Considering the mean heart rate, several studies [22, 27, 32, 33, 63] demonstrated a higher heart rate in healthy women as compared to age-matched healthy men. We could confirm these results by showing that the median NN-interval was significantly lower (a higher mean heart rate) in females in the youngest age group of 25–34 years. A trend for females of the next age groups (ranging from 35–74 years) seemed to be apparent in terms of a lower significance in comparison to age-related matched males. Furthermore, according to [20–22, 63] we could show that, independently of gender, the mean NN-interval did not differ significantly between the various age decades.

According to the linear ACOR analysis, no representative study using healthy subjects was found which investigates the effects of gender and age on the decay of the ACOR and of this function’s highest auxiliary maximum. In contrast to males, the NN interval time series of females are less predictable and more complex. An age-related increase of decay indices of the ACOR functions in both genders until an age of 45 years (as seen by the lower slope of the function courses; Kruskal-Wallis test) reflects an increasing predictability that can be interpreted as a further marker for reduced complexity with aging.

### 2 Frequency Domain

As reported in [39], directly comparing the values of frequency domain indices from various studies is quite difficult due to the different calculation modes (such as fast Fourier transform vs. autoregressive methods, different window functions, partly spectral corrections and others) and due to the units of the result values (e.g. ms2, natural or common logarithm). Our results showed that females had a slightly decreased absolute LF power in the age range of 25–44 years and a significantly increased absolute HF power between 35–54 years in comparison to male groups in the same age category confirming results from other studies [22, 24, 29, 30, 33, 63]. These gender differences disappeared at ages >44 years for LF and >54 years for HF. These results concur with other studies which described a disappearance of gender differences for the absolute LF and HF at ages <40 years [30, 33], <50 years [19] and < = 55 years [22].

Regarding the relative LF power (LF/P), a significant decrease in females compared to males for the age decades ranging from 25–54 years was determined. For ages 55 and higher, this gender-related effect disappeared. Independent of age, relative HF power (HF/P) and normalized HF power (HFn) were shown to be significantly higher; the normalized index LFn was found to be considerably lower in the female groups versus male groups. The studies performed by Agelink et al. and Beckers et al. [22, 33] confirm our observed differences with regard to the normalized frequency domain indices. Furthermore, the total power P only decreased in trend for females aged 35–74 years, also demonstrated in [3, 22, 28, 29, 64]. In comparison to males, LF/HF significantly decreased in females throughout all age decades. This observation was also reported in [16, 19, 22, 24].

With regard to age, the ratio LF/HF and the relative power LFn increased and the relative powers HF/P and HFn decreased in females aged 35–44 years when compared to younger females aged 25–34 years. These female age differences disappeared in ages over 44 years. For males, the increase of LF/HF and LFn and the decrease of HF/P and HFn disappeared somewhat later, namely at ages older than 54 years. However, Greiser et al.[24] and Stein et al. [65] published a graded inverse association with age in both genders for LF/HF. Both studies, however, considered only elderly subjects >44 years [24] and >65 years [65], respectively. We also found the trend of a median decrease for LF/HF in females aged 55–64 years when compared to the younger female group of 45–54 years, as well as in males aged 65–74 years when compared to the younger male group of 55–64 years. Rajendra Acharya et al. [38] even showed an LF/HF over age. The absolute power indices of LF and HF decreased with aging in both genders. However, the age-differences disappeared at the age of 65 years and older, confirming the results of several other studies [3, 16, 19–21, 27, 28, 63].

### 3 Nonlinear dynamics

Considering the nonlinear dynamics methods we can summarize that in short term analyses, nonlinear indices exhibit considerable age and gender dependencies (Tables 4, 6 and 8). With all measures, HRV complexity declines with aging. With increasing age, this influence decreases (Tables 5–8). Further, the differences in HRV complexity between men and women (Tables 9 and 10) decrease.

However, there are several specific observed differences when comparing indices that are derived from the different methods. When comparing YM with YF (Tables 3 and 4) we notice high significant differences in several indices from standard and short time symbolic dynamics which reflects a somewhat higher complexity in females (higher shannon_SD, fwrenyi025 and fwrenyi4, lower forbword and most of STSD indices) as opposed to males (Table 4). We obtained a similar result from the mutual information (AMI) where the decay of the curve is higher in females, suggesting a lower predictability due to higher complexity. This result is in accordance with similar findings of Ryan et al. [62] investigating 67 subjects (three age groups) and Pikkujamsa et al. [66] investigating 389 middle aged men and women applying approximate entropy. Interestingly, these complexity differences nearly disappear in the EM/EF comparison.

Further, DFA exhibits a significantly lower α1 (and a somewhat higher α2) in females than in males (both for YM/YF and EM/EF, though stronger in the group YM/YF). Even the values are all in the typical “healthy” region, being around one. This confirms the findings of Pikkujamsa et al. [66]. Nevertheless, one must consider that the application of DFA requires a much longer time series. This means that these results need to be treated with caution.

Traditional Poincaré analysis (SD1/SD2), the rows of segmented Poincaré analysis and, along with them, the partly correlated asymmetric patterns from IA indicate much stronger gender differences in the younger ages then in the elderly ones. The ratio SD1/SD2 decreases with aging, but remains significantly different between men and women. Considering IA indices that have some correlations with SPPA [50] (acceleration: r_7, r_8; deceleration: r_5, r_6) there is a lower number of decelerations and a higher number of accelerations (higher AS1-AS3) in YM as compared to YF. This indicates a significant gender difference due to an asymmetry in the Poincaré plot that disappears with aging. This gender dependency could be the reason for the more indifferent results found in IA indices and were published recently [39].

One problem of comparability with other studies is due to the lack of investigating gender dependencies in those studies. In the publication of Rajendra Acharya et al. [38], for example, there were partly significant differences (DFA, PPA) when compared to our findings. These differences were probably caused by the smoothing effect occurring when investigating mixed-sex populations.

### 4 General age and gender influences

An important finding of the present study is the occurrence of significant differences in many linear and nonlinear HRV indices when comparing the younger age groups, namely 25–34, 35–44 and 45–54 years, irrespective of gender. Between the age groups 45–54, 55–64 and 65–74 years, the number of significant different HRV indices and the significance values decreases drastically. Interestingly, the major age-related differences are between groups 2 and 3 in both genders (35–44 vs. 45–54 years). Comparing age groups 3 and 4 (45–54 vs. 55–64), we observe considerably higher differences in females than in males. Furthermore, the gender differences nearly disappear (exception FD) with aging. This is especially true for the age group 55–64 years and is probably at least in part caused by the influence of the menopause in women (and men). However, this is a point of controversy in scientific discussion. Snieder et al. [67] found in 196 male and 210 female middle-aged twins that oral contraceptive use and menopausal status had no effect on HRV. However, Moodithaya et al. [68] concluded that both aging and declined estrogen levels are associated with the autonomic alterations that are seen among postmenopausal women. Pikkujämsä et al. [4] demonstrated an increased baroreflex sensitivity and total HRV in postmenopausal women with estrogen replacement therapy compared to women without hormone therapy suggesting that hormonal factors play at least partly a role for the observed age- and gender-related differences. Additionally, women exhibit a higher vagal and a lower sympathetic modulation than men. This result is in accordance with Agelink et al. [22] and Britton et al. [64] who also found a higher LF power and lower HF power in young and middle aged men compared to age-matched women, suggesting a higher sympathetic activity and a lower parasympathetic tone in men. Huikuri et al. [69] also obtained this result. He found during the overshoot phase of the Valsalva maneuver, a lower heart rate response to a sudden rise in the blood pressure, a reduced normalized LF/P and a higher HF power in women compared to men reflecting a reduced baroreflex responsiveness and an increased tonic vagal activity in women.

The mechanism for gender differences in age-associated changes in cardiac autonomic function is obscure. Differences in the autonomic system may be due to differences in afferent receptor stimulation, in central reflex transmission, in the efferent nervous system, and in postsynaptic signaling. A few studies have indicated that female sex hormones influence autonomic modulation and estrogen has a facilitating effect on cardiac vagal function [27, 62]. Ryan et al. 1994 [62], assumed as a further possible source for a different heart rate dynamic between males and females some gender differences in baseline variables, such as blood pressure and associated alterations in the autonomic nervous system function. However, after adjustment for differences in baseline variables, Pikkujämsä et al. [4] suggested hormonal or genetic factors as more closely related to the mechanisms behind gender-related differences than gender-differences in lifestyle or laboratory values.

As proven in this study, from an age of 55 years onward, a distinction between the elderly age decades seems to play no essential role. An interesting aspect is that several indices of HRV (amongst others indices from the irreversibility analysis and SPPA-method) do not differ significantly when comparing age decades, especially when looking for gender differences. Here a clear age dependency can be found in men but marginally if at all for women. The gender dependency disappears for all indices (with the exception of FD) in the oldest groups (65–74 years) and for most indices already present in the age group of 55–64 years. This is also confirmed by the TD indices by Bonnemeier et al. [70]. For this reason, it can be supposed that starting from approximately age of 50 years onwards, a variety of HRV indices might have the potential as age- and gender independent risk markers for certain diseases. Nevertheless, considering the test for a general age and gender dependency (Tables 3–4) we must note that, besides FD, there is a remaining gender dependency in the meanNN (higher in males—meaning a lower heart rate), in α1 (higher in males), in SD1/SD2 (higher in females) and in ACOR/AMI (higher correlation values in females). The analysis of linear time- and frequency domain HRV indices confirmed the known reduction of HRV with population ageing. However, while the parasympathetic activity decreases with aging, the sympathetic one increases.

The age-dependent alteration of HRV indices is not surprising and is caused by modifications of the cardiovascular system with aging as found by Ferrari [71]. He stated amongst others that aging is accompanied by significant cardiovascular modifications, both structural as loss of sinoatrial pacemaker cells [5] or of arterial distensibility [6] and functional as altered coupling between regulatory components [5]. A slight degree of left ventricular hypertrophy develops with ageing, while the resting heart rate and early filling rate are somewhat decreased. Neurohumoral systems relevant to cardiovascular regulation are not uniformly affected by aging: the sympathetic nervous system is overactive and the circulating levels of vasopressin and atrial natriuretic factor are enhanced, while the activity of the renin-angiotensin system is blunted. It was further found by Fukusaki et al. [21] that age-related changes in HRV reflecting vagal modulation of heart rate were primarily mediated by aging per se and not by physiologic changes characteristic of normative aging. In a study of Porta et al. [72], comparing different age groups (21–30, 31–40, 41–50, 51–60 and 61–70 years) each consisting of 20 non-smoking healthy subjects, the complexity analysis was performed not only to NN interval time series but also to the respiration (RESP) and systolic arterial pressure (SAP). Besides the progressively decrease of the HRV complexity with aging during supine resting indicating an impairment of the cardiac regulation, with advancing age, he observed also (a) a gradually increase of HRV complexity during standing probably reflecting an impairment of the cardiac control and of the cardiac regulation response to stressors (b) a gradually decrease of SAP complexity during supine resting but not during standing suggesting a progressive increase of sympathetic activity and a reduced responsiveness of the vasomotor control to stressors, and (c) an age-unrelated RESP complexity indicating a preserved respiratory control in elderly healthy subjects. In contrast to our study, Porta et al. [72] also investigated the age dependency of the strength of the causal relations among the cardiovascular and the cardiorespiratory variability. Thereby it was noted that changes of the cardiovascular and the cardiorespiratory variability with ageing are probably linked with (a) a progressive efficiency loss of the baroreflex, (b) a progressive exploitation of the Frank-Starling mechanism under resting supine (c) an increase of the peripheral resistances during standing, (d) an increase of the sympathetic tone (e) a gradual uncoupling of the respiratory activity and the vagal outflow, (d) a progressive increase of the left ventricular thickness and of the vascular stiffness, and (e) a decrease of the respiratory sinus arrhythmia. Most of the previous attempts to explain the possible mechanisms leading to age and gender related HRV changes are more or less speculative and have be to be examined more extensively in physiological studies.

### 5 Importance of data pre-processing and stationarity

A remaining problem that exists when comparing results from different studies is the non-standardized signal pre-processing (upon request the author can supply the corresponding MATLAB-routine for pre-processing HRV time series to make the results more comparable). To make the comparison of results obtained in several studies more reliable, a general degree of standardization of the signal pre-processing and unified HRV analysis methods are required. For this purpose, each publication should include information about the reference studies of the applied methods and about the parameter settings used for the free parameters of each method. A further problem is the degree of reproducibility of HRV results in non-stationary time series. Variations in NN interval time series can be triggered mainly by the complex dynamics of the nonlinear system but also by diverse environmental factors [47] resulting most likely in a problem when differentiating these variations. The most linear methods of HRV analysis (e.g. Fourier transform) but also partially nonlinear methods require stationarity of the time series [73] which is problematic because most biosignals are non-stationary. To minimize this problem, in the present study, quasi-stationary NN interval time series were analyzed, acquired by following measures: accurate ECG recording was carried out under the same conditions, in a quit environment, and in resting supine position; and during the careful pre-processing all automatically detected NN intervals were visually controlled and corrected if necessary.

### 6 Summary

In the present representative study, for the first time, both age- and gender-related reference values for nonlinear short-term HRV indices were determined analyzing 5-min NN interval time series from a large cohort of 1906 healthy subjects. Here many nonlinear HRV indices were significantly dependent on the subjects’ age and gender. In addition to indices from traditional linear methods (of time- and frequency-domain) applicable for a general HRV quantification, HRV indices from nonlinear methods provide valuable information about the dynamics and the structure of NN interval time series. Nonlinear complexity measures of HRV based on entropy enable the quantification of irregularity in time series. In this study, decreasing entropy values with ageing were found denoting lower uncertainty levels and diminished complexity of the NN interval time series in elderly. In addition, it has been proven that women show a higher complexity of heart beat generation in the younger ages than men.

In summary, we found for almost all short-term HRV indices significant age dependencies. Whereas methods from TD, SD, STSD and correlation analyses (AMI, ACOR) showed the highest age dependencies, methods from IA and SPPA showed lowest age dependencies. Regarding to the age-dependent changes in short-term HRV indices between the five investigated age decades, a clear general age-dependent HRV with highest influences within the age groups 1 to 4 (25–54 years) was proven. The strongest aging effect could be obtained by comparing the subject groups 2 and 3. However, age-induced differences of HRV indices diminished with aging and were nearly disappeared with the exception of wsdwar from SD in males, between the two highest age groups 4 and 5 (55–64 and 65–74 years).

Regarding to the gender, significant gender dependencies were ascertained for approximately 50% of all calculated short-term HRV indices. Thus, there are lesser gender dependencies than age dependencies. The gender-dependent HRV differences start to disappear in age group 4 (exceptions are FD, α1, ST_OV, SD1/SD2, AMI, ACOR) and disappeared (with exception of FD) in age group 5. Nearly no gender differences could be proven in TD indices and CE indices. Finally, it has been noted that the aging effect is stronger in men than in women.

## Conclusion

In conclusion, it was noted that: (a) gender dependencies of several short-term HRV indices could be observed, (b) gender dependencies disappear (with exception of FD and a few indices that express weak significant differences) with an age older than 55 years, 10 years earlier than the disappearance of age dependencies, and (c) the behavior of age dependencies is different between males and females—more pronounced in men than in women.

The gender differences in the younger ages could probably be caused by the different hormonal situations leading to a higher sympathetic activity and a lower parasympathetic tone in men and vice versus in women. These gender differences disappear with aging presumably by the hormonal restructuring especially caused by the menopause in women but also in men leading to more comparable hormonal stages.

The age dependency of HRV could mainly be caused by significant cardiovascular modifications, both structural as loss of sinoatrial pacemaker cells or of arterial distensibility and functional as altered coupling between regulatory components. This leads to a considerably loss of variability and complexity.

Gender and age influences need to be considered when performing future HRV studies. This applies particularly to when major age and/or gender differences are present between study groups but also if these differences only partly differ. Furthermore, particularly in younger age groups it is mandatory to differentiate more strongly between the ages of subjects and/or patients due to considerably age- and gender-dependent differences within the younger age decades. Additionally, studies should investigate how far the capability of short-term HRV indices as indicators for cardiovascular diseases or as tool for preliminary diagnosis of certain autonomous regulation influencing diseases is dependent on age and gender influences. A subsequent study will consider the influence of heart diseases, social and lifestyle factors on HRV indices.

## Supporting Information

S1 Complementary Statistics Median values and interquartile ranges from all statistical tests. Table A: Descriptive statistics for linear HRV indices according to Tests I considering females and males each for two different age cluster 25–49 years and 50–74 years. Table B: Descriptive statistics for nonlinear HRV indices according to Tests I considering females and males each for two different age cluster 25–49 years and 50–74 years. Table C: Descriptive statistics for linear HRV indices according to Tests II considering female subjects divided into five different age decades 25–34, 35–44, 45–54, 55–64 and 65–74 years. Table D: Descriptive statistics for nonlinear HRV indices according to Tests II considering female subjects divided into five different age decades 25–34, 35–44, 45–54, 55–64 and 65–74 years. Table E: Descriptive statistics for linear HRV indices according to Tests II considering male subjects divided into five different age decades 25–34, 35–44, 45–54, 55–64 and 65–74 years. Table F: Descriptive statistics for nonlinear HRV indices according to Tests II considering male subjects divided into five different age decades 25–34, 35–44, 45–54, 55–64 and 65–74 years.
(DOCX)
