# Supplementary material for: Short-Term Heart Rate Variability—Influence of Gender and Age in Healthy Subjects
Source: PLoS One. 2015 Mar 30;10(3):e0118308. doi: 10.1371/journal.pone.0118308 (PMC4378923; doi:10.1371/journal.pone.0118308)
Supplement: S1 Complementary Statistics — Table A: Descriptive statistics for linear HRV indices according to Tests I considering females and males each for two different age cluster 25–49 years and 50–74 years. Table B: Descriptive statistics for nonlinear HRV indices according to Tests I considering females and males each for two different age cluster 25–49 years and 50–74 years. Table C: Descriptive statistics for linear HRV indices according to Tests II considering female subjects divided into five different age decades 25–34, 35–44, 45–54, 55–64 and 65–74 years. Table D: Descriptive statistics for nonlinear HRV indices according to Tests II considering female subjects divided into five different age decades 25–34, 35–44, 45–54, 55–64 and 65–74 years. Table E: Descriptive statistics for linear HRV indices according to Tests II considering male subjects divided into five different age decades 25–34, 35–44, 45–54, 55–64 and 65–74 years. Table F: Descriptive statistics for nonlinear HRV indices according to Tests II considering male subjects divided into five different age decades 25–34, 35–44, 45–54, 55–64 and 65–74 years. (DOCX) [file pone.0118308.s001.docx]

**Table A.** Descriptive statistics for linear HRV indices according to Tests I considering females and males each for two different age cluster 25-49 years and 50-74 years.

|  |  | **Median [lower quartile (0.25) - upper quartile (0.75)]** | | | |  |
| --- | --- | --- | --- | --- | --- | --- |
| **MA** | **HRV-index** | **YF** | **YM** | **EF** | **EM** | |
|  |  | **N=571** | **N=744** | **N=211** | **N=380** | |
| **TD** | meanNN | 898 [815-982] | 929 [830-1021] | 881 [811-949] | 909 [820-997] |  |
|  | sdNN | 41.0 [32.6-54.5] | 43.1 [33.2-54.0] | 29.0 [22.7-38.0] | 30.0 [22.7-40.2] |  |
|  | cvNN | 0.05 [0.04-0.06] | 0.05 [0.04-0.06] | 0.03 [0.03-0.04] | 0.03 [0.03-0.04] |  |
|  | sdaNN1 | 15.1 [10.8-21.8] | 15.6 [10.8-22.5] | 12.2 [8.6-18.2] | 12.8 [8.5-18.5] |  |
|  | rmssd | 31.6 [22.4-45.4] | 30.2 [21.5-42.6] | 18.9 [13.3-27.7] | 17.9 [12.8-25.9] |  |
|  | pNN50 | 10.3 [2.5-2.8] | 8.8 [2.2-2.3] | 1.2 [0-6.0] | 1.2 [0-5.4] |  |
|  | pNNl20 | 45.8 [31.5-63.6] | 49.5 [34.5-66.6] | 73.8 [54.7-88.8] | 76.0 [59.7-90.7] |  |
|  | renyi4 | 3.86 [3.52-4.24] | 3.89 [3.52-4.23] | 3.33 [2.98-3.63] | 3.39 [3.00-3.78] |  |
|  | shannon_h | 4.29 [3.98-4.68] | 4.34 [3.99-4.66] | 3.80 [3.47-4.13] | 3.85 [3.45-4.24] |  |
| **FD** | LF | 100 [55-186] | 133 [70-235] | 38 [23-76] | 51 [26-102] |  |
|  | HF | 72.0 [32.5-159.3] | 60.7 [29.0-123.3] | 21.8 [10.6-48.4] | 18.2 [8.0-36.5] |  |
|  | P | 362 [209-630] | 384 [220-665] | 162 [89-278] | 186 [101-347] |  |
|  | LF/HF | 1.43 [0.77-2.58] | 2.27 [1.29-4.22] | 1.91 [0.98-3.48] | 3.03 [1.81-5.43] |  |
|  | LF/P | 0.29 [0.21-0.40] | 0.36 [0.25-0.49] | 0.27 [0.18-0.35] | 0.30 [0.20-0.42] |  |
|  | HF/P | 0.21 [0.12-0.33] | 0.16 [0.09-0.25] | 0.15 [0.08-0.24] | 0.10 [0.05-0.16] |  |
|  | LFn | 0.59 [0.44-0.72] | 0.69 [0.56-0.81] | 0.66 [0.49-0.78] | 0.75 [0.64-0.84] |  |
|  | HFn | 0.41 [0.28-0.56] | 0.31 [0.19-0.44] | 0.34 [0.22-0.51] | 0.25 [0.16-0.36] |  |

Abbreviations: YF, young females; YM, young males; EF, elderly females; EM, elderly males; MA, method of analysis; TD, time domain; FD, frequency domain.**Table B.** Descriptive statistics for nonlinear HRV indices according to Tests I considering females and males each for two different age cluster 25-49 years and 50-74 years.

|  |  | **Median [lower quartile (0.25) - upper quartile (0.75)]** | | | |  |
| --- | --- | --- | --- | --- | --- | --- |
| **MA** | **HRV-index** | **YF** | **YM** | **EF** | **EM** | |
|  |  | **N=571** | **N=744** | **N=211** | **N=380** | |
| **SD** | shannon_SD | 3.13 [2.74-3.42] | 3.02 [2.70-3.34] | 2.46 [2.17-2.84] | 2.42 [2.07-2.77] |  |
|  | forbword | 27 [19-35] | 30 [22-36] | 39 [32-44] | 39 [35-44] |  |
|  | wpsum02 | 0.49 [0.28-0.70] | 0.53 [0.35-0.69] | 0.78 [0.63-0.89] | 0.77 [0.61-0.90] |  |
|  | wpsum13 | 0.06 [0.03-0.12] | 0.07 [0.03-0.13] | 0.03 [0.01-0.07] | 0.04 [0.01-0.08] |  |
|  | wsdvar | 1.21 [0.93-1.57] | 1.23 [0.95-1.51] | 0.81 [0.54-1.08] | 0.85 [0.51-1.18] |  |
|  | phvar5 | 0.45 [0.30-0.59] | 0.42 [0.27-0.57] | 0.22 [0.09-0.35] | 0.2 [0.09-0.33] |  |
|  | plvar20 | 0.01 [0-0.07] | 0.01 [0-0.12] | 0.19 [0.03-0.58] | 0.25 [0.06-0.59] |  |
|  | fwrenyi025 | 3.46 [3.19-3.69] | 3.39 [3.15-3.64] | 2.99 [2.80-3.29] | 3.01 [2.75-3.22] |  |
|  | fwrenyi4 | 2.52 [2.01-2.93] | 2.36 [1.97-2.80] | 1.75 [1.40-2.11] | 1.67 [1.38-2.06] |  |
| **DFA** | α1 | 0.92 [0.76-1.07] | 0.98 [0.83-1.14] | 1.07 [0.86-1.24] | 1.14 [0.99-1.28] |  |
|  | α2 | 0.90 [0.78-1.06] | 0.88 [0.72-1.02] | 1.00 [0.85-1.09] | 0.96 [0.83-1.11] |  |
| **CE** | H_c_^3,3^ | 0.78 [0.72-0.84] | 0.78 [0.72-0.83] | 0.68 [0.62-0.75] | 0.67 [0.61-0.74] |  |
| **STSD** | ST_MP | 0.70 [0.65-0.75] | 0.73 [0.68-0.77] | 0.75 [0.70-0.79] | 0.76 [0.71-0.80] |  |
|  | ST_0V | 0.05 [0-0.10] | 0 [0-0.09] | 0 [0-0.08] | 0 [0-0] |  |
|  | ST_1V | 0.44 [0.38-0.53] | 0.49 [0.42-0.61] | 0.53 [0.43-0.64] | 0.55 [0.43-0.66] |  |
|  | ST_2V | 0.28 [0.21-0.34] | 0.24 [0.17-0.32] | 0.20 [0.11-0.27] | 0.18 [0.11-0.28] |  |
|  | ST_INC | 0.08 [0.06-0.11] | 0.08 [0.05-0.11] | 0.06 [0.03-0.09] | 0.06 [0.03-0.10] |  |
|  | ST_DESC | 0.08 [0.05-0.10] | 0.07 [0.04-0.10] | 0.05 [0.03-0.08] | 0.06 [0.03-0.09] |  |
|  | ST_PEAK | 0.14 [0.11-0.18] | 0.13 [0.10-0.16] | 0.14 [0.10-0.18] | 0.14 [0.10-0.18] |  |
|  | ST_VAL | 0.16 [0.13-0.20] | 0.14 [0.10-0.17] | 0.16 [0.13-0.19] | 0.14 [0.11-0.18] |  |
|  | ST_2LV | 0.16 [0.12-0.20] | 0.16 [0.11-0.21] | 0.11 [0.08-0.17] | 0.12 [0.07-0.18] |  |
|  | ST_2UV | 0.31 [0.25-0.37] | 0.27 [0.21-0.33] | 0.29 [0.24-0.36] | 0.29 [0.23-0.35] |  |
| **PPA** | SD1 | 22.3 [15.8-32.1] | 21.3 [15.2-30.2] | 13.4 [9.5-19.5] | 12.7 [9.0-18.3] |  |
|  | SD2 | 52.0 [42.4-69.3] | 56.4 [43.1-70.3] | 38.2 [28.9-50.1] | 40.4 [30.1-54.2] |  |
|  | SD1/SD2 | 0.42 [0.34-0.54] | 0.39 [0.31-0.48] | 0.34 [0.26-0.45] | 0.31 [0.25-0.40] |  |
| **SPPA** | SPPA_c_4 | 2.05 [1.24-2.97] | 1.94 [1.16-2.84] | 1.94 [1.25-2.84] | 2.17 [1.33-3.02] |  |
|  | SPPA_c_5 | 12.4 [10.4-14.6] | 12.4 [10.3-14.4] | 12.1 [9.3-14.3] | 12.1 [9.7-14.5] |  |
|  | SPPA_c_6 | 34.7 [31.7-37.4] | 34.9 [31.7-37.6] | 34.6 [30.6-37.5] | 33.6 [30.8-37.2] |  |
|  | SPPA_c_7 | 34.5 [31.7-38.4] | 34.6 [31.7-38.2] | 35.5 [32.4-39.4] | 35.1 [32.6-39.8] |  |
|  | SPPA_c_8 | 13.5 [12.0-14.8] | 13.1 [11.6-14.8] | 13.2 [11.1-14.7] | 13.3 [11.8-15.0] |  |
|  | SPPA_c_9 | 1.82 [1.01-2.60] | 1.81 [1.05-2.63] | 1.82 [0.86-2.60] | 1.57 [0.60-2.50] |  |
|  | SPPA_r_4 | 1.68 [1.00-2.35] | 2.02 [1.35-2.68] | 1.77 [1.14-2.41] | 1.88 [1.30-2.39] |  |
|  | SPPA_r_5 | 13.5 [11.8-15.3] | 12.5 [10.8-14.3] | 12.8 [10.9-14.5] | 12.2 [10.0-14.0] |  |
|  | SPPA_r_6 | 35.8 [32.6-38.1] | 34.6 [32.1-37.2] | 34.7 [31.9-38.2] | 35.9 [33.4-38.2] |  |
|  | SPPA_r_7 | 31.8 [28.5-35.3] | 34.9 [31.3-38.1] | 34.7 [31.5-38.4] | 35.0 [32.2-38.3] |  |
|  | SPPA_r_8 | 15.3 [13.2-17.2] | 13.8 [12.1-16.0] | 13.5 [11.6-15.4] | 12.8 [10.7-14.8] |  |
|  | SPPA_r_9 | 1.64 [1.07-2.18] | 1.49 [0.94-2.02] | 1.69 [1.13-2.18] | 1.72 [1.22-2.34] |  |
|  | SPPA_entropy | 4.04 [3.99-4.08] | 4.03 [3.97-4.08] | 4.03 [3.96-4.08] | 4.03 [3.94-4.09] |  |
| **IA** | AS1 | 49.0 [47.2-51.6] | 50.7 [48.5-52.6] | 50.3 [48.2-52.4] | 50.0 [48.4-51.9] |  |
|  | AS2 | 50.5 [47.4-54.7] | 52.9 [49.3-57.5] | 51.5 [48.1-58.3] | 51.7 [48.4-58.1] |  |
|  | AS3 | 0.005 [-0.008-0.030] | 0.015 [-0.001-0.049] | 0.008 [-0.008-0.071] | 0.008 [-0.007-0.071] |  |
| **ACOR and AMI** | a21rr | -2.40 [-2.61-(-2.18)] | -2.27 [-2.49-(-2.05)] | -2.17 [-2.39-(-1.91)] | -2.10 [-2.31-(-1.83)] |  |
|  | a31rr | -1.33 [-1.40-(-1.24)] | -1.29 [-1.37-(-1.20)] | -1.23 [-1.31-(-1.13)] | -1.21 [-1.30-(-1.10)] |  |
|  | x2peakrr | 5 [4-7] | 6 [5-8] | 6 [5-8] | 7 [5-9] |  |
|  | y2peakrr | 0.43 [0.36-0.52] | 0.40 [0.33-0.47] | 0.46 [0.38-0.60] | 0.43 [0.35-0.56] |  |
|  | amax21rr | -0.50 [-0.59-(-0.40)] | -0.46 [-0.56-(-0.35)] | -0.41 [-0.50-(-0.31)] | -0.37 [-0.48-(-0.28)] |  |
|  | a21rrcor | -0.30 [-0.45-(-0.21)] | -0.26 [-0.38-(-0.17)] | -0.21 [-0.34-(-0.13)] | -0.18 [-0.28-(-0.12)] |  |
|  | a31rrcor | -0.30 [-0.40-(-0.21)] | -0.27 [-0.36-(-0.20)] | -0.21 [-0.30-(-0.14)] | -0.19 [-0.25-(-0.13)] |  |
|  | x2peakrrcor | 5 [4-8] | 7 [4-10] | 6 [4-11] | 9 [4-13] |  |
|  | y2peakrrcor | 0.43 [0.30-0.54] | 0.36 [0.23-0.47] | 0.41 [0.24-0.57] | 0.34 [0.18-0.48] |  |
|  | amax21rrcor | -0.11 [-0.14-(-0.08)] | -0.09 [-0.14-(-0.06)] | -0.08 [-0.11-(-0.06)] | -0.08 [-0.11-(-0.05)] |  |

Abbreviations: YF, young females; YM, young males; EF, elderly females; EM, elderly males; MA, method of analysis; SD, symbolic dynamic; DFA, detrended fluctuation analysis; CE, compression entropy; STSD, short-term symbolic dynamic; PPA, Poincaré plot analysis; SPPA, segmented Poincaré plot analysis; IA, irreversibility analysis; ACOR and AMI, auto-correlation and auto-mutual information.

**Table C.** Descriptive statistics for linear HRV indices according to Tests II considering female subjects divided into five different age decades 25-34, 35-44, 45-54, 55-64 and 65-74 years.

|  |  | **25-34 years N=208** | **35-44 years N=259** | **45-54 years N=158** | **55-64 years N=95** | **65-74 years N=62** |
| --- | --- | --- | --- | --- | --- | --- |
| **MA** | **HRV-index** | **Median [lower quartile (0.25) - upper quartile (0.75)]** | | | | |
| **TD** | meanNN | 893 [818-975] | 903 [807-994] | 901 [830-976] | 847 [782-956] | 881 [814-923] |
|  | sdNN | 44.9 [35.4-58.1] | 40.2 [32.9-53.7] | 35.5 [26.5-44.5] | 29.0 [22.8-35.2] | 26.0 [18.7-32.5] |
|  | cvNN | 0.05 [0.04-0.06] | 0.05 [0.04-0.06] | 0.04 [0.03-0.05] | 0.03 [0.03-0.04] | 0.03 [0.02-0.04] |
|  | sdaNN1 | 15.0 [10.8-21.4] | 16.0 [10.6-23.3] | 14.9 [10.6-20.8] | 11.2 [7.8-17.5] | 10.5 [8.0-15.4] |
|  | rmssd | 38.4 [26.1-54.5] | 30.5 [22.4-45.7] | 23.7 [16.9-31.3] | 18.9 [13.2-27.3] | 16.9 [11.1-23.9] |
|  | pNN50 | 18.3 [4.9-40.3] | 9.3 [2.1-28.4] | 3.4 [0.5-9.5] | 1.1 [0-6.0] | 0.5 [0-4.6] |
|  | pNNl20 | 37.6 [26.4-55.8] | 47.2 [32.5-63.8] | 62.5 [45.1-75.8] | 74.1 [54.7-89.2] | 81.9 [64.2-93.9] |
|  | renyi4 | 4.01 [3.65-4.36] | 3.85 [3.54-4.26] | 3.57 [3.25-3.93] | 3.31 [2.99-3.57] | 3.13 [2.78-3.55] |
|  | shannon_h | 4.43 [4.11-4.77] | 4.28 [3.98-4.66] | 4.05 [3.70-4.38] | 3.79 [3.49-4.00] | 3.65 [3.20-3.95] |
| **FD** | LF | 116 [68-216] | 100 [56-198] | 69 [37-140] | 36 [23-72] | 30 [14-56] |
|  | HF | 105 [52-217] | 72 [31-147] | 40 [19-78] | 20 [10-46] | 15 [8-29] |
|  | P | 444 [251-727] | 352 [217-654] | 264 [152-437] | 156 [82-239] | 125 [65-226] |
|  | LF/HF | 1.14 [0.63-2.09] | 1.49 [0.78-2.64] | 1.92 [0.99-3.43] | 1.91 [1.06-3.45] | 1.93 [0.89-3.93] |
|  | LF/P | 0.29 [0.21-0.40] | 0.29 [0.20-0.42] | 0.26 [0.20-0.36] | 0.30 [0.20-0.36] | 0.26 [0.17-0.32] |
|  | HF/P | 0.26 [0.16-0.38] | 0.20 [0.11-0.32] | 0.15 [0.09-0.23] | 0.15 [0.08-0.24] | 0.12 [0.07-0.25] |
|  | LFn | 0.53 [0.39-0.68] | 0.60 [0.44-0.73] | 0.66 [0.50-0.77] | 0.66 [0.51-0.78] | 0.66 [0.47-0.80] |
|  | HFn | 0.47 [0.32-0.61] | 0.40 [0.27-0.56] | 0.34 [0.23-0.50] | 0.34 [0.22-0.49] | 0.34 [0.20-0.53] |

Abbreviations: MA, method of analysis; TD, time domain; FD, frequency domain.

**Table D.** Descriptive statistics for nonlinear HRV indices according to Tests II considering female subjects divided into five different age decades 25-34, 35-44, 45-54, 55-64 and 65-74 years.

|  |  | **25-34 years N=208** | **35-44 years N=259** | **45-54 years N=158** | **55-64 years N=95** | **65-74 years N=62** |
| --- | --- | --- | --- | --- | --- | --- |
| **MA** | **HRV-index** | **Median [lower quartile (0.25) - upper quartile (0.75)]** | | | | |
| **SD** | shannon_SD | 3.31 [2.99-3.63] | 3.09 [2.70-3.40] | 2.75 [2.40-3.08] | 2.44 [2.18-2.84] | 2.27 [1.94-2.56] |
|  | forbword | 23 [14-31] | 28 [20-35] | 35 [28-40] | 39 [32-44] | 42 [35-46] |
|  | wpsum02 | 0.41 [0.23-0.58] | 0.50 [0.29-0.68] | 0.68 [0.48-0.81] | 0.79 [0.65-0.88] | 0.83 [0.73-0.93] |
|  | wpsum13 | 0.07 [0.03-0.13] | 0.07 [0.03-0.12] | 0.05 [0.02-0.09] | 0.03 [0.01-0.06] | 0.02 [0.01-0.05] |
|  | wsdvar | 1.36 [1.05-1.65] | 1.22 [0.93-1.56] | 1.01 [0.72-1.34] | 0.81 [0.56-1.07] | 0.67 [0.40-0.95] |
|  | phvar5 | 0.54 [0.37-0.64] | 0.44 [0.30-0.58] | 0.31 [0.18-0.46] | 0.22 [0.09-0.39] | 0.13 [0.04-0.31] |
|  | plvar20 | 0 [0-0.02] | 0.01 [0-0.07] | 0.06 [0.01-0.22] | 0.24 [0.03-0.59] | 0.33 [0.07-0.74] |
|  | fwrenyi025 | 3.62 [3.35-3.83] | 3.42 [3.19-3.67] | 3.19 [2.99-3.44] | 2.97 [2.81-3.29] | 2.90 [2.57-3.16] |
|  | fwrenyi4 | 2.79 [2.29-3.16] | 2.47 [2.01-2.87] | 2.06 [1.72-2.51] | 1.72 [1.41-2.10] | 1.51 [1.28-1.81] |
| **DFA** | α1 | 0.87 [0.69-1.00] | 0.95 [0.79-1.09] | 1.02 [0.85-1.20] | 1.06 [0.87-1.21] | 1.09 [0.86-1.31] |
|  | α2 | 0.88 [0.76-1.01] | 0.91 [0.79-1.07] | 0.95 [0.82-1.08] | 0.99 [0.85-1.08] | 0.98 [0.82-1.12] |
| **CE** | H_c_^3,3^ | 0.82 [0.75-0.86] | 0.78 [0.72-0.84] | 0.73 [0.68-0.78] | 0.68 [0.62-0.74] | 0.64 [0.59-0.70] |
| **STSD** | ST_MP | 0.69 [0.62-0.74] | 0.71 [0.65-0.76] | 0.73 [0.69-0.78] | 0.75 [0.70-0.79] | 0.76 [0.70-0.79] |
|  | ST_0V | 0.06 [0-0.11] | 0.04 [0-0.10] | 0 [0-0.09] | 0 [0-0.09] | 0 [0-0.02] |
|  | ST_1V | 0.43 [0.37-0.49] | 0.45 [0.39-0.53] | 0.48 [0.40-0.60] | 0.52 [0.43-0.64] | 0.54 [0.40-0.66] |
|  | ST_2V | 0.30 [0.22-0.35] | 0.28 [0.20-0.34] | 0.23 [0.16-0.30] | 0.20 [0.11-0.28] | 0.19 [0.10-0.29] |
|  | ST_INC | 0.08 [0.06-0.11] | 0.08 [0.06-0.11] | 0.07 [0.04-0.10] | 0.06 [0.03-0.09] | 0.06 [0.03-0.08] |
|  | ST_DESC | 0.08 [0.06-0.10] | 0.08 [0.05-0.10] | 0.07 [0.05-0.10] | 0.05 [0.03-0.08] | 0.05 [0.02-0.08] |
|  | ST_PEAK | 0.14 [0.11-0.18] | 0.14 [0.11-0.18] | 0.14 [0.11-0.17] | 0.15 [0.11-0.18] | 0.14 [0.10-0.18] |
|  | ST_VAL | 0.17 [0.13-0.21] | 0.16 [0.12-0.19] | 0.16 [0.12-0.19] | 0.15 [0.12-0.20] | 0.16 [0.13-0.20] |
|  | ST_2LV | 0.15 [0.13-0.21] | 0.16 [0.12-0.20] | 0.14 [0.09-0.20] | 0.11 [0.07-0.16] | 0.10 [0.07-0.16] |
|  | ST_2UV | 0.31 [0.26-0.37] | 0.31 [0.25-0.36] | 0.29 [0.25-0.35] | 0.30 [0.24-0.36] | 0.30 [0.25-0.38] |
| **PPA** | SD1 | 27.0 [18.5-38.5] | 21.6 [15.8-32.5] | 16.8 [11.9-22.2] | 13.4 [9.3-19.3] | 11.6 [7.8-16.4] |
|  | SD2 | 56.4 [44.9-72.6] | 52.2 [42.4-69.7] | 46.3 [35.5-58.1] | 36.8 [28.9-46.9] | 32.7 [24.1-43.5] |
|  | SD1/SD2 | 0.47 [0.37-0.60] | 0.41 [0.33-0.51] | 0.35 [0.29-0.45] | 0.34 [0.26-0.48] | 0.34 [0.25-0.43] |
| **SPPA** | SPPA_c_4 | 1.92 [1.35-2.83] | 2.19 [1.19-3.06] | 2.01 [1.05-2.88] | 2.16 [1.39-3.00] | 1.76 [1.11-2.68] |
|  | SPPA_c_5 | 12.3 [10.7-14.6] | 12.4 [10.5-14.5] | 12.6 [9.4-14.4] | 11.8 [9.0-13.8] | 12.9 [10.1-15.9] |
|  | SPPA_c_6 | 35.5 [32.6-37.8] | 34.0 [31.4-37.2] | 34.5 [31.2-36.9] | 34.2 [30.4-37.5] | 34.6 [30.7-38.4] |
|  | SPPA_c_7 | 33.5 [31.0-36.6] | 35.2 [31.9-38.9] | 35.6 [32.8-39.6] | 35.8 [32.4-39.7] | 34.7 [31.6-39.4] |
|  | SPPA_c_8 | 13.6 [12.2-15.1] | 13.6 [12.0-14.7] | 12.9 [11.3-14.5] | 13.6 [10.7-14.5] | 13.3 [11.9-15.2] |
|  | SPPA_c_9 | 1.97 [1.24-2.68] | 1.69 [0.90-2.51] | 1.67 [0.98-2.63] | 1.61 [0.63-2.55] | 2.06 [1.12-2.53] |
|  | SPPA_r_4 | 1.62 [0.97-2.28] | 1.71 [0.96-2.42] | 1.75 [1.10-2.45] | 1.85 [1.24-2.51] | 1.63 [0.98-2.24] |
|  | SPPA_r_5 | 13.9 [12.3-15.7] | 13.2 [11.6-15.3] | 13.1 [11.6-14.8] | 12.7 [10.7-14.4] | 12.3 [10.3-14.3] |
|  | SPPA_r_6 | 35.9 [32.3-38.1] | 35.7 [32.5-38.1] | 35.3 [32.5-37.7] | 34.4 [31.5-38.8] | 36.1 [32.4-38.5] |
|  | SPPA_r_7 | 31.1 [27.5-34.5] | 32.1 [28.7-36.0] | 33.2 [29.8-36.1] | 34.8 [31.3-38.4] | 35.2 [32.3-39.2] |
|  | SPPA_r_8 | 15.7 [14.1-17.9] | 15.1 [12.9-16.9] | 14.5 [12.6-16.1] | 13.6 [11.6-15.8] | 12.4 [9.9-14.4] |
|  | SPPA_r_9 | 1.55 [0.99-2.13] | 1.70 [1.10-2.26] | 1.64 [1.11-2.14] | 1.65 [1.04-2.07] | 1.83 [1.26-2.39] |
|  | SPPA_entropy | 4.05 [4.00-4.08] | 4.03 [3.99-4.07] | 4.04 [3.97-4.08] | 4.03 [3.96-4.08] | 4.03 [3.96-4.08] |
| **IA** | AS1 | 48.8 [46.9-51.5] | 49.1 [47.3-51.6] | 49.5 [47.4-51.7] | 50.5 [47.8-52.5] | 50.3 [48.8-52.5] |
|  | AS2 | 50.8 [47.3-54.0] | 50.5 [47.7-55.0] | 51.1 [47.3-56.0] | 51.6 [49.2-56.8] | 51.5 [47.7-61.1] |
|  | AS3 | 0.005 [-0.007-0.027] | 0.005 [-0.009-0.033] | 0.005 [-0.009-0.039] | 0.009 [-0.005-0.055] | 0.010 [-0.010-0.133] |
| **ACOR and AMI** | a21rr | -2.48 [-2.70-(-2.28)] | -2.39 [-2.57-(-2.15)] | -2.26 [-2.44-(-2.01)] | -2.18 [-2.44-(-1.91)] | -2.04 [-2.34-(-1.82)] |
|  | a31rr | -1.35 [-1.42-(-1.28)] | -1.32 [-1.40-(-1.23)] | -1.28 [-1.35-(-1.17)] | -1.23 [-1.32-(-1.13)] | -1.21 [-1.29-(-1.07)] |
|  | x2peakrr | 5 [4-6] | 5 [4-7] | 6 [5-8] | 6 [5-8] | 6 [5-9] |
|  | y2peakrr | 0.44 [0.36-0.51] | 0.44 [0.36-0.53] | 0.44 [0.36-0.53] | 0.48 [0.37-0.60] | 0.46 [0.39-0.61] |
|  | amax21rr | -0.54 [-0.62-(-0.45)] | -0.50 [-0.58-(-0.39)] | -0.44 [-0.53-(-0.32)] | -0.42 [-0.49-(-0.30)] | -0.36 [-0.50-(-0.30)] |
|  | a21rrcor | -0.36 [-0.53-(-0.24)] | -0.30 [-0.42-(-0.21)] | -0.23 [-0.34-(-0.16)] | -0.21 [-0.37-(-0.13)] | -0.22 [-0.31-(-0.12)] |
|  | a31rrcor | -0.35 [-0.46-(-0.25)] | -0.29 [-0.39-(-0.21)] | -0.24 [-0.33-(-0.17)] | -0.21 [-0.30-(-0.15)] | -0.19 [-0.27-(-0.12)] |
|  | x2peakrrcor | 4 [4-6] | 5 [4-8] | 5 [4-9] | 6 [4-10] | 6 [4-13] |
|  | y2peakrrcor | 0.44 [0.32-0.53] | 0.42 [0.29-0.55] | 0.42 [0.31-0.52] | 0.42 [0.24-0.59] | 0.40 [0.22-0.56] |
|  | amax21rrcor | -0.12 [-0.16-(-0.09)] | -0.11 [-0.14-(-0.07)] | -0.09 [-0.13-(-0.07)] | -0.09 [-0.11-(-0.06)] | -0.08 [-0.12-(-0.05)] |

Abbreviations: MA, method of analysis; SD, symbolic dynamic; DFA, detrended fluctuation analysis; CE, compression entropy; STSD, short-term symbolic dynamic; PPA, Poincaré plot analysis; SPPA, segmented Poincaré plot analysis; IA, irreversibility analysis; ACOR and AMI, auto-correlation and auto-mutual information.**Table E.** Descriptive statistics for linear HRV indices according to Tests II considering male subjects divided into five different age decades 25-34, 35-44, 45-54, 55-64 and 65-74 years.

|  |  | **25-34 years N=330** | **35-44 years N=292** | **45-54 years N=235** | **55-64 years N=183** | **65-74 years N=84** |
| --- | --- | --- | --- | --- | --- | --- |
| **MA** | **HRV-index** | **Median [lower quartile (0.25) - upper quartile (0.75)]** | | | | |
| **TD** | meanNN | 945 [845-1030] | 923 [816-1007] | 908 [833-1014] | 908 [817-986] | 908 [816-980] |
|  | sdNN | 46.0 [36.4-58.9] | 42.6 [32.3-53.1] | 34.5 [26.1-44.3] | 29.9 [22.0-39.3] | 26.5 [20.1-35.4] |
|  | cvNN | 0.05 [0.04-0.06] | 0.04 [0.04-0.06] | 0.04 [0.03-0.05] | 0.03 [0.03-0.04] | 0.03 [0.02-0.04] |
|  | sdaNN1 | 15.5 [10.9-22.8] | 16.1 [11.1-23.3] | 14.8 [9.7-20.3] | 13.1 [8.2-18.2] | 11.0 [7.4-18.0] |
|  | rmssd | 36.2 [26.2-48.5] | 28.7 [20.9-39.0] | 21.5 [15.7-29.6] | 17.5 [12.5-24.8] | 16.7 [11.8-23.4] |
|  | pNN50 | 15.3 [5.3-30.3] | 6.8 [1.9-18.8] | 2.1 [0.3-8.4] | 1.1 [0-3.7] | 0.8 [0-4.8] |
|  | pNNl20 | 39.7 [30.0-57.1] | 51.8 [38.4-67.8] | 68.8 [50.7-82.9] | 77.7 [60.5-91.2] | 83.0 [69.4-92.4] |
|  | renyi4 | 4.00 [3.69-4.35] | 3.86 [3.52-4.18] | 3.58 [3.23-3.99] | 3.37 [2.97-3.77] | 3.26 [2.91-3.54] |
|  | shannon_h | 4.45 [4.13-4.78] | 4.33 [3.97-4.63] | 4.04 [3.68-4.40] | 3.83 [3.43-4.22] | 3.70 [3.29-4.06] |
| **FD** | LF | 160 [83-258] | 133 [67-238] | 78 [41-140] | 45 [26-97] | 43 [18-72] |
|  | HF | 88 [43-161] | 54 [27-100] | 25 [11-51] | 17 [8-37] | 11 [6-25] |
|  | P | 429 [271-764] | 392 [212-646] | 266 [129-461] | 176 [96-334] | 157 [85-271] |
|  | LF/HF | 1.93 [1.12-3.42] | 2.43 [1.37-4.70] | 2.98 [1.76-5.19] | 3.08 [1.80-5.50] | 3.06 [1.74-5.25] |
|  | LF/P | 0.38 [0.27-0.49] | 0.37 [0.25-0.51] | 0.33 [0.22-0.44] | 0.29 [0.21-0.40] | 0.26 [0.16-0.42] |
|  | HF/P | 0.19 [0.12-0.28] | 0.15 [0.09-0.23] | 0.11 [0.06-0.18] | 0.10 [0.05-0.16] | 0.08 [0.05-0.16] |
|  | LFn | 0.66 [0.53-0.77] | 0.71 [0.58-0.82] | 0.75 [0.64-0.84] | 0.75 [0.64-0.85] | 0.75 [0.63-0.84] |
|  | HFn | 0.34 [0.23-0.47] | 0.29 [0.18-0.42] | 0.25 [0.16-0.36] | 0.25 [0.15-0.36] | 0.25 [0.16-0.37] |

Abbreviations: MA, method of analysis; TD, time domain; FD, frequency domain.

**Table F.** Descriptive statistics for nonlinear HRV indices according to Tests II considering male subjects divided into five different age decades 25-34, 35-44, 45-54, 55-64 and 65-74 years.

|  |  | **25-34 years N=330** | **35-44 years N=292** | **45-54 years N=235** | **55-64 years N=183** | **65-74 years N=84** |
| --- | --- | --- | --- | --- | --- | --- |
| **MA** | **HRV-index** | **Median [lower quartile (0.25) - upper quartile (0.75)]** | | | | |
| **SD** | shannon_SD | 3.19 [2.93-3.45] | 2.96 [2.67-3.26] | 2.65 [2.29-2.96] | 2.42 [2.06-2.75] | 2.27 [1.94-2.56] |
|  | forbword | 25 [19-33] | 31 [24-37] | 37 [31-42] | 39 [35-44] | 41 [37-46] |
|  | wpsum02 | 0.45 [0.28-0.60] | 0.56 [0.37-0.70] | 0.70 [0.52-0.83] | 0.77 [0.62-0.89] | 0.83 [0.72-0.92] |
|  | wpsum13 | 0.08 [0.04-0.14] | 0.07 [0.04-0.13] | 0.05 [0.02-0.11] | 0.04 [0.01-0.07] | 0.02 [0.01-0.06] |
|  | wsdvar | 1.32 [1.04-1.58] | 1.21 [0.96-1.50] | 1.02 [0.69-1.32] | 0.88 [0.53-1.11] | 0.68 [0.44-0.95] |
|  | phvar5 | 0.51 [0.35-0.62] | 0.39 [0.25-0.54] | 0.27 [0.13-0.41] | 0.19 [0.07-0.32] | 0.17 [0.06-0.29] |
|  | plvar20 | 0 [0-0.04] | 0.02 [0-0.14] | 0.13 [0.02-0.39] | 0.27 [0.07-0.62] | 0.40 [0.14-0.64] |
|  | fwrenyi025 | 3.54 [3.31-3.72] | 3.35 [3.15-3.56] | 3.12 [2.86-3.36] | 3.00 [2.75-3.18] | 2.90 [2.61-3.06] |
|  | fwrenyi4 | 2.57 [2.18-2.97] | 2.28 [1.94-2.71] | 1.92 [1.49-2.27] | 1.66 [1.36-2.01] | 1.49 [1.24-1.80] |
| **DFA** | α1 | 0.92 [0.79-1.06] | 1.00 [0.85-1.15] | 1.12 [0.97-1.26] | 1.15 [1.02-1.3] | 1.15 [0.98-1.28] |
|  | α2 | 0.86 [0.67-1.00] | 0.86 [0.72-0.99] | 0.98 [0.80-1.09] | 0.94 [0.84-1.08] | 1.01 [0.89-1.17] |
| **CE** | H_c_^3,3^ | 0.80 [0.75-0.85] | 0.77 [0.72-0.82] | 0.71 [0.65-0.77] | 0.68 [0.60-0.74] | 0.65 [0.59-0.70] |
| **STSD** | ST_MP | 0.70 [0.66-0.76] | 0.74 [0.68-0.77] | 0.76 [0.70-0.80] | 0.76 [0.72-0.81] | 0.77 [0.70-0.80] |
|  | ST_0V | 0.03 [0-0.11] | 0 [0-0.07] | 0 [0-0.02] | 0 [0-0] | 0 [0-0] |
|  | ST_1V | 0.47 [0.41-0.57] | 0.50 [0.42-0.61] | 0.54 [0.44-0.65] | 0.57 [0.45-0.66] | 0.56 [0.42-0.69] |
|  | ST_2V | 0.26 [0.19-0.32] | 0.24 [0.18-0.32] | 0.20 [0.11-0.28] | 0.17 [0.11-0.26] | 0.18 [0.08-0.28] |
|  | ST_INC | 0.09 [0.06-0.11] | 0.09 [0.06-0.12] | 0.07 [0.04-0.10] | 0.06 [0.03-0.10] | 0.05 [0.02-0.08] |
|  | ST_DESC | 0.07 [0.05-0.10] | 0.08 [0.05-0.10] | 0.06 [0.04-0.09] | 0.06 [0.03-0.09] | 0.05 [0.02-0.08] |
|  | ST_PEAK | 0.14 [0.11-0.16] | 0.13 [0.10-0.17] | 0.13 [0.10-0.17] | 0.14 [0.10-0.18] | 0.15 [0.11-0.19] |
|  | ST_VAL | 0.15 [0.11-0.18] | 0.13 [0.10-0.16] | 0.14 [0.10-0.18] | 0.14 [0.12-0.17] | 0.16 [0.11-0.20] |
|  | ST_2LV | 0.16 [0.13-0.20] | 0.18 [0.12-0.21] | 0.13 [0.08-0.19] | 0.12 [0.07-0.17] | 0.11 [0.05-0.17] |
|  | ST_2UV | 0.28 [0.23-0.34] | 0.26 [0.21-0.32] | 0.28 [0.22-0.34] | 0.28 [0.23-0.34] | 0.30 [0.24-0.37] |
| **PPA** | SD1 | 25.6 [18.5-34.3] | 20.4 [14.8-27.6] | 15.2 [11.1-21.0] | 12.4 [8.8-17.4] | 11.6 [8.2-16.5] |
|  | SD2 | 59.1 [47.6-76.6] | 56.0 [42.1-70.4] | 46.4 [34.9-60.2] | 40.6 [29.6-52.6] | 35.4 [27.3-46.7] |
|  | SD1/SD2 | 0.42 [0.34-0.52] | 0.37 [0.29-0.45] | 0.32 [0.25-0.40] | 0.31 [0.25-0.40] | 0.30 [0.25-0.44] |
| **SPPA** | SPPA_c_4 | 1.97 [1.22-2.78] | 1.84 [1.06-2.74] | 2.14 [1.29-3.10] | 2.17 [1.27-2.91] | 2.37 [1.51-3.15] |
|  | SPPA_c_5 | 12.5 [10.5-14.8] | 12.6 [10.3-14.7] | 11.9 [10.0-14.2] | 11.8 [9.5-14.4] | 12.0 [9.3-14.3] |
|  | SPPA_c_6 | 35.0 [32.1-37.2] | 34.7 [31.5-37.8] | 34.5 [31.5-37.2] | 33.6 [31.0-37.6] | 33.9 [30.2-37.6] |
|  | SPPA_c_7 | 34.4 [31.5-37.7] | 34.4 [31.7-38.3] | 35.2 [31.6-39.3] | 35.3 [33.1-39.8] | 35.7 [32.5-40.5] |
|  | SPPA_c_8 | 13.2 [11.7-14.6] | 13.2 [11.2-14.9] | 13.1 [11.8-15.1] | 13.2 [11.6-14.9] | 13.5 [11.9-15.8] |
|  | SPPA_c_9 | 1.85 [1.15-2.60] | 1.83 [1.04-2.81] | 1.56 [0.78-2.62] | 1.56 [0.57-2.43] | 1.35 [0.35-2.20] |
|  | SPPA_r_4 | 2.03 [1.46-2.70] | 2.01 [1.32-2.64] | 1.92 [1.23-2.51] | 1.92 [1.28-2.48] | 1.84 [1.31-2.30] |
|  | SPPA_r_5 | 12.5 [11.0-14.5] | 12.6 [10.7-14.2] | 12.6 [10.4-14.2] | 12.2 [10.3-13.5] | 11.1 [8.6-14.0] |
|  | SPPA_r_6 | 34.5 [31.7-37.1] | 34.5 [32.0-37.2] | 35.2 [33.1-37.7] | 35.4 [33.5-38.1] | 36.7 [33.6-39.7] |
|  | SPPA_r_7 | 34.4 [31.0-38.3] | 35.1 [31.1-38.2] | 35.2 [31.9-37.5] | 34.9 [32.3-37.9] | 35.8 [32.5-39.2] |
|  | SPPA_r_8 | 14.4 [12.5-16.4] | 13.7 [11.9-15.7] | 13.2 [11.3-15.2] | 12.7 [10.6-14.4] | 12.3 [9.0-14.9] |
|  | SPPA_r_9 | 1.43 [0.83-1.93] | 1.46 [1.00-2.16] | 1.66 [1.18-2.22] | 1.64 [1.26-2.35] | 1.89 [1.16-2.38] |
|  | SPPA_entropy | 4.05 [3.98-4.08] | 4.03 [3.97-4.08] | 4.05 [3.96-4.09] | 4.02 [3.93-4.08] | 4.01 [3.89-4.08] |
| **IA** | AS1 | 51.0 [48.5-53.1] | 50.6 [48.5-52.6] | 50.0 [48.0-51.9] | 50.2 [48.5-51.6] | 50.3 [48.6-52.0] |
|  | AS2 | 53.3 [49.7-57.9] | 53.0 [49.2-57.7] | 51.3 [48.0-56.1] | 51.7 [48.1-57.9] | 53.5 [49.1-61.3] |
|  | AS3 | 0.017 [0.001-0.051] | 0.016 [-0.001-0.050] | 0.006 [-0.008-0.038] | 0.007 [-0.007-0.065] | 0.025 [-0.004-0.116] |
| **ACOR and AMI** | a21rr | -2.37 [-2.57-(-2.14)] | -2.23 [-2.45-(-2.03)] | -2.11 [-2.32-(-1.87)] | -2.08 [-2.25-(-1.82)] | -2.11 [-2.30-(-1.78)] |
|  | a31rr | -1.33 [-1.39-(-1.24)] | -1.28 [-1.37-(-1.20)] | -1.22 [-1.31-(-1.12)] | -1.20 [-1.28-(-1.09)] | -1.18 [-1.28-(-1.07)] |
|  | x2peakrr | 5 [4-7] | 6 [5-8] | 7 [5-9] | 7 [5-10] | 7 [5-9] |
|  | y2peakrr | 0.39 [0.33-0.47] | 0.40 [0.33-0.49] | 0.42 [0.35-0.52] | 0.41 [0.34-0.54] | 0.44 [0.36-0.57] |
|  | amax21rr | -0.49 [-0.58-(-0.40)] | -0.45 [-0.55-(-0.36)] | -0.38 [-0.50-(-0.29)] | -0.36 [-0.47-(-0.26)] | -0.37 [-0.48-(-0.28)] |
|  | a21rrcor | -0.31 [-0.42-(-0.21)] | -0.24 [-0.34-(-0.17)] | -0.18 [-0.27-(-0.12)] | -0.17 [-0.28-(-0.12)] | -0.18 [-0.32-(-0.12)] |
|  | a31rrcor | -0.31 [-0.40-(-0.23)] | -0.26 [-0.34-(-0.19)] | -0.20 [-0.27-(-0.14)] | -0.18 [-0.24-(-0.13)] | -0.17 [-0.24-(-0.13)] |
|  | x2peakrrcor | 6 [4-9] | 7 [4-11] | 8 [5-11] | 9 [5-15] | 8 [4-14] |
|  | y2peakrrcor | 0.36 [0.24-0.47] | 0.35 [0.23-0.48] | 0.36 [0.20-0.47] | 0.34 [0.15-0.48] | 0.34 [0.15-0.48] |
|  | amax21rrcor | -0.10 [-0.16-(-0.07)] | -0.09 [-0.13-(-0.06)] | -0.08 [-0.11-(-0.06)] | -0.07 [-0.11-(-0.05)] | -0.07 [-0.11-(-0.05)] |

Abbreviations: MA, method of analysis; SD, symbolic dynamic; DFA, detrended fluctuation analysis; CE, compression entropy; STSD, short-term symbolic dynamic; PPA, Poincaré plot analysis; SPPA, segmented Poincaré plot analysis; IA, irreversibility analysis; ACOR and AMI, auto-correlation and auto-mutual information.
